# Effect of dietary soybean saponin Bb on the growth performance, intestinal nutrient absorption, morphology, microbiota, and immune response in juvenile Chinese soft-shelled turtle (*Pelodiscus sinensis*)

**DOI:** 10.3389/fimmu.2022.1093567

**Published:** 2022-12-23

**Authors:** Yue Wang, Xinyue Jia, Zixue Guo, Ling Li, Tianyu Liu, Peiyu Zhang, Haiyan Liu

**Affiliations:** ^1^ Laboratory of Aquatic Animal Nutrition and Ecology, College of Life Sciences, Hebei Normal University, Shijiazhuang, China; ^2^ Key Laboratory of Animal Physiology, Biochemistry and Molecular Biology of Hebei Province, Shijiazhuang, China

**Keywords:** Soybean saponin Bb, growth performance, macronutrients and minerals absorption, intestine morphology, gut microbiota, intestine health, *Pelodiscus sinensis*

## Abstract

Soybean meal is widely applied in the aquafeeds due to the limitation of fish meal resources. Numerous studies have manifested that dietary soybean saponin, an anti-nutrient factor in soybean meal, may slow growth and induce intestinal inflammation in aquatic animals, but the possible causes are unclear. The juvenile *Pelodiscus sinensis* (mean initial body weight: 6.92 ± 0.03 g) were fed basal diet (CON group) and 2.46% soybean saponin Bb-supplemented diet (SAP group) for 35 days to further explore the effects of dietary soybean saponin Bb on the growth performance, apparent digestibility coefficients, intestinal morphology, the gut microbiota, intestinal transporters/channels, and immune-related gene expression. The results indicated that dietary soybean saponin Bb significantly decreased final body weight, specific growth rate, protein deposition ratio, and apparent digestibility coefficients (dry matter, crude protein, and crude lipid) of nutrients in *Pelodiscus sinensis*, which may be closely correlated with markedly atrophic villus height and increased lamina propria width in the small intestine. In addition, plasma contents of cholesterol, calcium, phosphorus, potassium, lysozyme, and C3 were significantly decreased in the SAP group compared with the control group. Soybean saponin Bb significantly downregulated the mRNA levels of glucose transporter 2, fatty acid binding protein 1 and fatty acid binding protein 2, amino acid transporter 2, b^0,+^-type amino acid transporter 1, and sodium-dependent phosphate transport protein 2b in the small intestine. At the same time, the expressions of key transcription factors (*STAT1*, *TBX21*, *FOS*), chemokines (*CCL3*), cytokines (*TNF-α*, *IL-8*), and aquaporins (*AQP3*, *AQP6*) in the inflammatory response were increased by soybean saponin Bb in the large intestine of a turtle. Additionally, dietary supplementation of SAP significantly reduced the generic abundance of beneficial bacteria (*Lactobacillus*, *Bifidobacterium*, and *Bacillus*) and harmful bacteria (*Helicobacter* and *Bacteroides*). In a nutshell, dietary supplementation of 2.46% soybean saponin not only hindered the growth performance by negatively affecting the macronutrients absorption in the small intestine but also induced an inflammatory response in the large intestine possibly by damaging the intestinal morphology, disturbing the intestinal microbiota and decreasing intestinal epithelial cell membrane permeability.

## 1 Introduction

The Chinese soft-shelled turtle (*Pelodiscus sinensis*) has a high edible value ascribing to its relatively high protein content, unsaturated fatty acids, and essential trace elements (1) and has become a significant freshwater-cultured species in China with an annual production of more than 330,000 tons (2). As a carnivorous omnivore, the turtle has a comparatively high protein requirement, which is mainly supplied by white fishmeal in the diet. In view of the high price and low availability of white fishmeal, searching for suitable fishmeal alternative protein sources or manufacturing low-fishmeal diets has become a major concern for researchers and formulators in the feed industry. Soybean meal (SBM) is a common fishmeal substitute in aquafeed due to its steady source and relatively balanced amino acid profile (3, 4). However, high doses of dietary soybean meal supplementation led to an obvious decrease in growth rate and feed utilization efficiency in Atlantic salmon (*Salmo salar*) (5), rainbow trout (*Oncorhynchus mykiss*) (6), turbot (*Scophthalmus maximus*) (7), zebrafish (*Danio rerio*) (8), and grass carp (*Ctenopharyngodon idella*) (9). In addition, long-term feeding of soybean meal also resulted in structural and functional changes in the distal intestine, including shortened mucosal fold height, widened lamina propria, impaired absorptive function, and a profound infiltration of inflammatory cells in the lamina propria (5), which were commonly referred to as “soybean meal-induced inflammation.” This negative influence may be correlated with antinutritional factors in soybean meal such as β-conglycinin, glycinin, trypsin inhibitor, isoflavone, saponins, etc. (10).

Although the antinutritional factors responsible for the aforementioned adverse effects have not been specified, some alcohol-soluble components in full-fat soybean meal (11) are likely to be to blame. It was because soy protein concentrate obtained through ethanol extraction technology did not induce pathological changes in the distal intestine of Atlantic salmon, whereas full-fat soybean meal caused intestinal inflammation in Atlantic salmon (12). Among the alcohol-soluble components, soybean saponins are polycyclic triterpene glycosides, which have been proposed as inducers of intestinal inflammation in some fish, such as zebrafish (13) and turbot (14). Additionally, saponins had relatively good heat stability (15), making passivation by extrusion processing technology during aquafeed manufacture or other heat treatments difficult. Furthermore, when the soybean saponins enter the alimentary tract, their amphipathic might interfere with membrane homeostasis, increase the permeability of cell membranes, and result in the uptake of antigens and potential toxins (16, 17). They are divided into group A, group B, group E, and DDMP based on their linked sugar side chains, and the saponin Bb is the most abundant component among the saponin groups (18). Therefore, soybean saponin Bb could be used as a representative compound to study the biological effects on aquatic animals. Several studies have shown that soybean saponin reduced weight gain, changed intestinal morphology, increased the permeability of enterocyte membrane (13), and induced upregulation of proinflammatory factors (*IL-1β*, *IL-8*, *TNF-α*) in aquatic animals such as Japanese flounder (*Paralichthys olivaceus*) (19), Atlantic salmon (20, 21), turbot (7, 14, 22), rice field eel (*Monopterus albus*) (23), zebrafish (24), and gilthead sea bream (*Sparus aurata*) (11). However, further studies are needed to investigate the effect of soybean saponin Bb on the absorption and transport of nutrients in aquatic animals and the variation tendency of intestinal key pattern recognition receptors, transcription factors, and cellular signaling pathways that regulate the inflammatory response.

The present work was designed to explore the effects of 2.46% soybean saponin Bb in the diet on growth, nutrient digestion and absorption, intestinal morphology, gut microbiota, and intestinal immune signaling pathway, and reveal the possible reasons for the reduction of growth performance and the underlying inflammatory process in Chinese soft-shelled turtle. This work will contribute to a more comprehensive understanding of the adverse effects of dietary soy saponin in aquaculture animals.

## 2 Materials and methods

### 2.1 Experimental diet and design

Fish meal, gelatin, and casein were used as main protein sources, and soybean oil and soybean lecithin were applied as primary lipid sources. By adding different levels of soybean saponin Bb (0%, 12%) purchased from Shaanxi Xiazhou Biotechnology Co. Ltd, two isonitrogenous (47% crude protein) and isoenergetic (5% crude lipid) experimental diets were prepared. The actual contents of saponin Bb in two diets measured by high-performance liquid chromatography (Shanghai Microspectrum Detection Technology Co. Ltd., Shanghai, China) were 0% (CON) and 2.46% (SAP), respectively (**Table 1**). Y_2_O_3_ was added as an external indicator to determine the apparent digestibility of each nutrient. Betaine was supplemented in two diets to increase diet palatability. All ingredients were step-by-step mixed, ground sufficiently, and sifted (80 mesh), then 4% of soybean oil was mixed thoroughly with the powder and passed through an 80-mesh sieve three times. The mixture was then homogenized thoroughly with distilled water (15% of the powder mass) in a blender to form a stiff dough. The dough was then extruded into soft pellets with a 2.0-mm diameter using a pelletizer (Youyi Machinery Factory), and the pellets were preserved in airtight plastic bags at −20°C until use. The diet formulation and its proximate chemical composition are shown in **Table 1**.

**Table 1:** Diet formulation and proximate chemical composition of the experimental diets (dry matter basis, g/kg).

Ingredients	CON	SAP
Russian fish meal	380.00	380.00
Casein	150.00	150.00
Gelatin	110.00	110.00
Squid liver meal	40.00	40.00
Soybean oil	40.00	40.00
Soybean lecithin	10.00	10.00
Microcrystalline cellulose	120.00	0.00
Soybean saponin Bb	0.00	120.00
Monocalcium phosphate	15.00	15.00
Limestone	8.00	8.00
Zeolite powder	50.00	50.00
Potassium chloride	2.00	2.00
Sodium chloride	1.00	1.00
Betaine	1.00	1.00
Choline chloride	2.00	2.00
Y_2_O_3_	1.00	1.00
Premix^a^	20.00	20.00
Carboxymethylcellulose sodium	20.00	20.00
Proximate composition		
Crude protein	47.91	47.09
Crude lipid	5.39	5.27
Crude ash	23.16	22.05
Gross energy (MJ/kg)	17.47	18.48
Soybean saponins Bb	0.00	2.46

a Premix was according to Zhang et al. (25).

### 2.2 Turtles and feeding management

**Figure 1:**
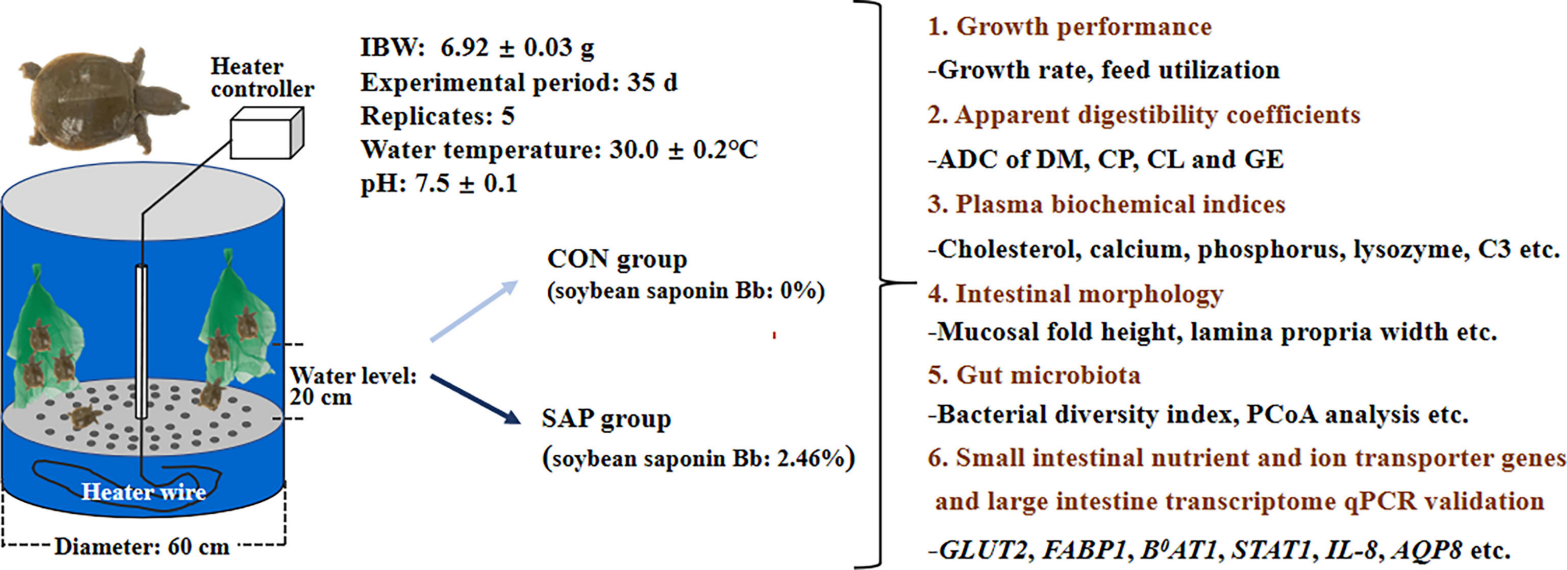
Flow chart of experimental design.

The flow chart of experimental design is shown in **Figure 1**. Chinese soft-shelled turtles, purchased from Tangshan, Hebei province, were acclimated for 10 days with juvenile turtle commercial feed in the Laboratory of Physiology and Ecology, Hebei Normal University. In total, 70 juvenile turtles with an initial body weight of 6.92 ± 0.03 g (mean carapace length: 26.77 mm) were evenly assigned to 10 tanks (250 L) with hanging nets for shelter 24 h after starvation, and each of the five tanks was randomly fed same experimental diet for 5 weeks. The juvenile turtles were fed twice a day (8:30, 17:30), and the residual pellets were siphoned out 40 min after each meal. Approximately one-third of the tank’s water was renewed once a day in the morning. The aquaculture room was kept dark throughout the feeding trial except when the residual pellets/feces were removed and tank water was exchanged. The water temperature was maintained at 30°C ± 0.2°C using an independent temperature controller, and the pH was measured at 7.5 ± 0.1.

### 2.3 Sample collection

At the end of the feeding trial, juvenile turtles in each tank were bulk-weighed to determine the growth-related parameters. Two turtles were randomly selected, weighed, and stored at −20°C after 24 h of food deprivation to analyze the turtle body composition. The remaining turtles were anesthetized with 60 mg/L eugenol and weighed individually 3 h after the last meal. The visceral mass, liver, and limb fat of eight turtles per group were dissected and weighed to calculate the morphological parameters. Jugular venous blood was collected into an anticoagulant tube containing heparin sodium and centrifuged at 4,000×*g* at 4°C for 15 min to obtain plasma, and stored at −80°C for the determination of biochemical parameters.

The digestive tracts of the remaining 24 turtles in each group were dissected from the body cavity and stripped of mesentery. The intestinal contents from 10 turtles per group were collected in five cryopreserved tubes, which were quickly put into liquid nitrogen for freezing and stored at −80°C prior to the determination of the intestinal microbiota. Subsequently, the digestive tract is divided into the large and small intestines according to the division of the Chinese soft-shelled turtle intestine (26). The middle sections of the large and small intestines from eight turtles per group were taken at 1–2 cm and stored in 4% paraformaldehyde separately for histological evaluation. Finally, the large and small intestines of another 16 turtles were frozen in liquid nitrogen, then they were transferred to −80°C for subsequent intestine transcriptome and gene expression analysis.

### 2.4 Biochemical analysis

A previous study (25) was used to estimate the chemical composition of feed, turtle body, and feces. The contents of plasma glucose (A154-1-1), triglyceride (A110-1-1), total cholesterol (A111-1-1), total amino acid (A026-1-1), lysozyme (A050-1-1), complement 3 (E032-1-1), and IgM (E025-1-1) were determined by the colorimetric method using commercial diagnostic kits according to the manufacturer’s protocols (Nanjing Jiancheng Bioengineering Institute, Nanjing, Jiangsu, China). The plasma phosphorus (BC1650), calcium (BC0720), potassium (BC2770), magnesium (BC2790), zinc (BC2810), and sodium (BC2800) were also determined colorimetrically by mercantile kits (Beijing Solarbio Science & Technology Co. Ltd., Beijing, China).

### 2.5 Intestinal histology

The fixed large and small intestines of one turtle from each tank were sent to Wuhan Sevell Technology Co. Ltd. and processed according to standard histological techniques, and stained with hematoxylin and eosin (H&E). Sections were examined with a Leica Microsystems CMS GmBH Ernst-Leitz-Str. 17-37, 35578 (Wetzlar, Germany).

### 2.6 Intestinal microbiota

#### 2.6.1 DNA extraction and 16S rRNA gene sequencing

Intestinal chyme was collected from four turtles in each group. Microbial community genomic DNA was extracted from gut content samples using DNeasy PowerSoil Pro Kit (QIAGEN, USA) according to the manufacturer’s instructions. The DNA extract was checked on 1% agarose gel, and DNA concentration and purity were determined with NanoDrop 2000 UV-vis spectrophotometer (Thermo Scientific, Wilmington, USA).

The hypervariable region V3–V4 of the bacterial 16S rRNA gene was amplified with primer pairs 338F: ACTCCTACGGGAGGCAGCAG_806R: GGACTACHVGGGTWTCTAAT by an ABI GeneAmp^®^ 9700 PCR thermocycler (ABI, CA, USA). PCR reactions were performed in triplicate. The PCR product was extracted from 2% agarose gel and purified using the AxyPrep DNA Gel Extraction Kit (Axygen Biosciences, Union City, CA, USA) according to the manufacturer’s instructions and quantified using Quantus™ Fluorometer (Promega, USA). Purified amplicons were pooled in equimolar and paired-end sequenced on an Illumina MiSeq PE300 platform (Illumina, San Diego, CA, USA) according to the standard protocols by Majorbio Bio-Pharm Technology Co. Ltd. (Shanghai, China).

#### 2.6.2 Bioinformatics analysis

The raw 16S rRNA gene sequencing reads were demultiplexed, quality-filtered by fastp version 0.20.0, and merged by FLASH version 1.2.7 Operational taxonomic units (OTUs) with 97% similarity cutoff were clustered using UPARSE version 7.1, and chimeric sequences were identified and removed. The taxonomy of each OTU representative sequence was analyzed by RDP Classifier version 2.2 against the 16S rRNA database using a confidence threshold of 0.7. PCoA analysis was evaluated by similarity analysis (ANOSIM) based on the Bray-Curtis distance. All-against-all (more strict) comparison of Discriminant Analysis Effect Size (LEfSe) was also utilized using rarefied 16S rRNA gene sequence data, the cut-off logarithmic Linear Discriminant Analysis (LDA) score was 2.0.

### 2.7 RNA-Seq

Total RNA was extracted from the large intestines of the CON and SAP groups using TRIzol^®^ Reagent according to the manufacturer’s instructions (Invitrogen), and genomic DNA was removed using DNase I (TaKara). RNA-seq transcriptome librarly was prepared and the paired-end RNA-seq sequencing library was sequenced with the Illumina HiSeqxten/NovaSeq 6000 sequencer (2×150 bp read length).

The raw data (raw reads) obtained by the Illumina platform were treated with a series of quality control processes to obtain high-quality quality control data (clean reads). The clean reads were then aligned with the *Pelodiscus sinensis* genome (GCF_000230535.1 https://www.ncbi.nlm.nih.gov/genome/?term=Pelodiscus+sinensis), and they were spliced and *de novo* assembled using StringTie (https://ccb.jhu.edu/software/stringtie/index.shtml?t=example).

Differentially expressed genes (DEGs), GO function enrichment, and KEGG pathway analysis were the same as Jiang et al. (27). To validate the RNA-Seq results, 20 DEGs were selected for QPCR. The method is described in Calculation formula and Statistical analysis.

### 2.8 RNA extraction and real-time PCR

Total RNA was extracted from the small and large intestines by the TransZol Up method. The quality control of RNA, reverse transcription of mRNA to cDNA, primer design, PCR amplification procedure, assessment of PCR product, and quantitative real-time PCR protocol were performed referring to a previous study (25). 18RPS was applied in the small intestine, and β-actin was applied in the large intestine as housekeeping genes to normalize expressions of target genes. The expression levels of these genes were computed based on the 2^−ΔΔCt^ method. Primer sequence, product length, annealing temperature, and accession number are presented in **Supplementary Table S1**.

### 2.9 Calculation formula and statistical analysis

The formulas of growth index, body index, and nutrient apparent digestibility are shown in **Supplementary Table S2**.

All data were analyzed using statistics 10.0 (Statsoft Inc., Tulsa, OK, USA). The *t*-test method was used to analyze the two groups of data and described as mean ± SD. *p* < 0.05 was considered a significant difference.

## 3 Results

### 3.1 Growth performance

The growth performance of juvenile turtles in the current feeding trial is shown in **Table 2**. Compared with the CON group, the final body weight (FBW), weight gain rate (WGR), specific growth rate (SGR), feed conversion ratio (FCR), protein efficiency ratio (PER), protein deposition ratio (PDR), fat deposition ratio (FDR), and fatsomatic index (FSI) in the SAP group were significantly reduced, while feeding rate (FR) and FCR in the SAP group showed the opposite trend (*p* < 0.05). Survival rate (SR), viscerosomatic index (VSI), and hepatosomatic index (HSI) had no significant differences between the two groups (*p* > 0.05).

**Table 2:** Effects of dietary soybean saponin Bb on growth performance and morphology of juvenile *P. sinensis* (*n* = 5 for growth performance, *n* = 8 for morphological parameters).

Parameters	CON	SAP	*p*-value
SR (%)	100.00 ± 0.00	97.14 ± 0.39	0.347
IBW (g)	6.93 ± 0.03	6.92 ± 0.03	0.798
FBW (g)	17.49 ± 0.79	12.73 ± 1.57	< 0.001
FR (%/day)	3.66 ± 0.09	4.73 ± 0.28	< 0.001
WGR (%)	152.48 ± 10.48	83.95 ± 23.13	< 0.001
SGR (%/day)	2.64 ± 0.12	1.72 ± 0.35	0.001
FCR	1.48 ± 0.07	2.98 ± 0.46	< 0.001
PER	1.41 ± 0.07	0.73 ± 0.14	< 0.001
PDR (%)	67.62 ± 2.44	37.47 ± 6.13	< 0.001
FDR (%)	203.09 ± 6.34	127.94 ± 11.71	< 0.001
VSI (%)	10.93 ± 0.78	10.84 ± 1.77	0.855
HSI (%)	3.98 ± 0.39	4.00 ± 0.52	0.921
FSI (%)	3.40 ± 0.12	2.74 ± 0.35	< 0.001

SR, survival ratio; IBW, initial body weight; FBW, final body weight; FR, feeding ratio; WGR, weight gain ratio; SGR, specific growth ratio; FCR, feed conversion ratio; PER, protein efficiency ratio; PDR, protein deposition ratio; FDR, fat deposition ratio; VSI, viscerosomatic index; HSI, hepatosomatic index; FSI, fatsomatic index.

### 3.2 Proximate composition of the turtle’s whole body, apparent digestibility coefficients, and plasma biochemical indexes

**Figure 2:**
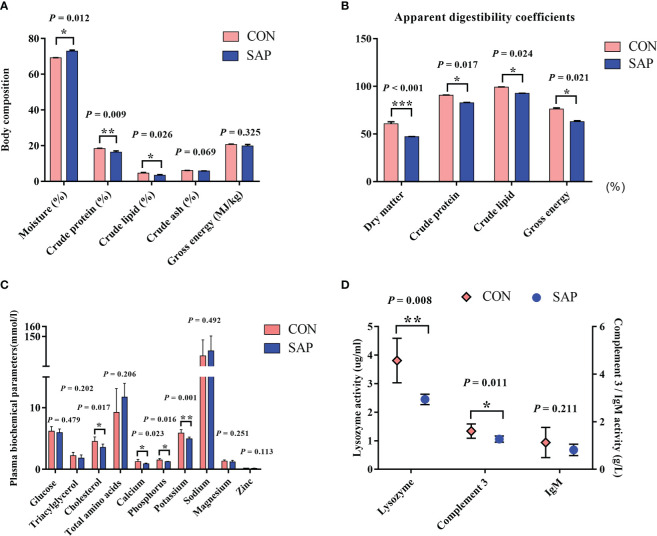
Effects of dietary soybean saponin Bb on body composition **(A)** (*n* = 3), apparent digestibility coefficients **(B)** (*n* = 3), plasma biochemical parameters **(C, D)** in juvenile *P. sinensis* (*n* = 6). ^*^ *p* < 0.05; ^**^ *p* < 0.01; ^***^ *p* < 0.001; Student’s *t*-test.

**Figure 2A** shows that the contents of moisture, crude protein, and crude lipid in the SAP group were significantly lower than those in the CON group (*p* < 0.05). **Figure 2B** indicates that the dietary soybean saponin Bb significantly decreased the apparent digestibility coefficients of dietary nutrients (dry matter, crude protein, and crude lipid) in juvenile turtles (*p* < 0.05). **Figure 2C** displays that the plasma cholesterol concentration of juvenile turtles in the SAP diet significantly decreased (*p* < 0.05), and glucose and triglyceride decreased but had no significant influence (*p* > 0.05). The concentration of serum calcium, phosphorus, and potassium in the SAP diet was significantly lower than those in the CON diet (*p* < 0.05). **Figure 2D** shows that lysozyme and C3 activities were significantly reduced in the SAP diet (*p* < 0.05).

### 3.3 Intestinal morphology

**Figure 3:**
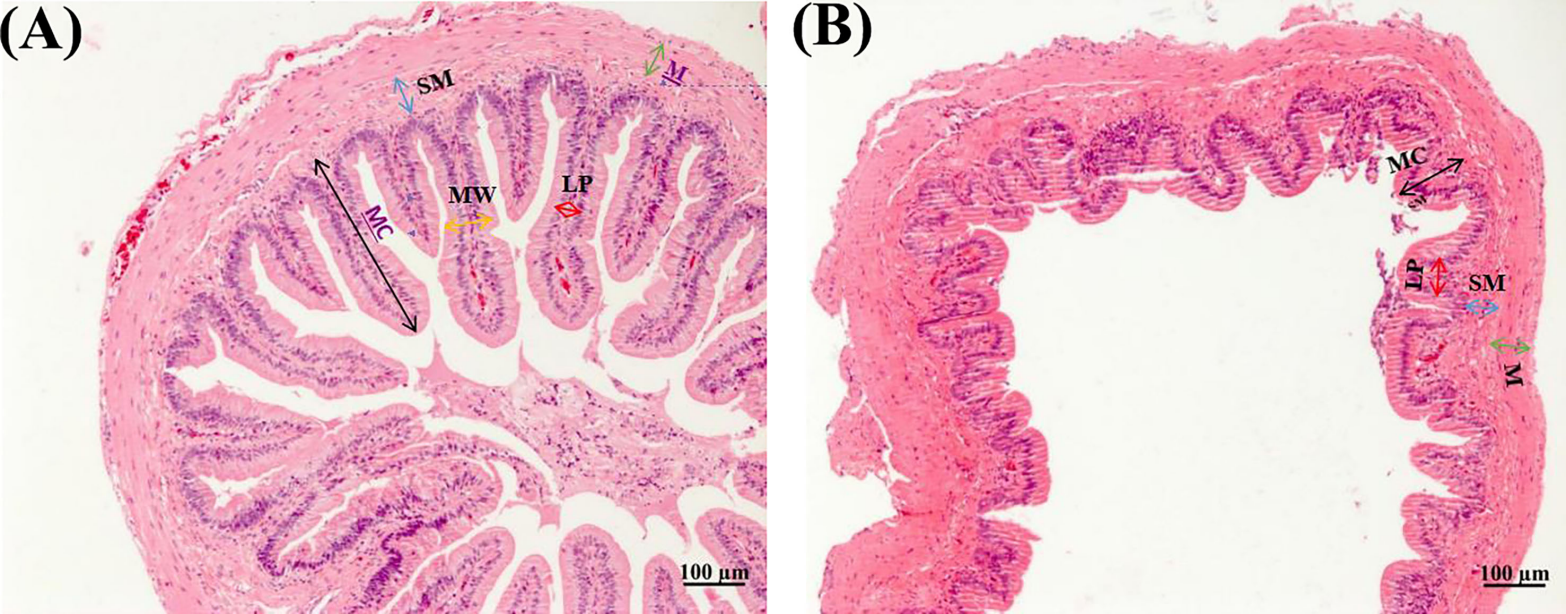
Morphology of small **(A)** and large **(B)** intestines of juvenile *P. sinensis* (×100).

**Table 3** shows that dietary soybean saponin Bb had remarkably decreased the mucosal fold height and increased lamina propria width of the small intestine (*p* < 0.05). However, in the large intestine, dietary soybean saponin Bb not only the mucosal fold height and lamina propria width the same change as in the small intestine but also the muscularis thickness was decreased (*p* < 0.05). The measurement range of intestinal microstructure parameters is shown in **Figure 3**.

**Table 3:** Effect of dietary soybean saponin Bb on the intestinal microstructure of juvenile *P. sinensis* (*n* = 3).

Parameters	CON	SAP	*p*-value
Small intestine			
Mucosal fold height (μm)	344.38 ± 9.46	281.80 ± 24.45	0.014
Mucosal fold width (μm)	103.32 ± 20.36	115.10 ± 17.66	0.449
Lamin propria width (μm)	14.85 ± 2.29	19.04 ± 1.17	0.048
Muscularis (μm)	66.77 ± 2.86	70.67 ± 5.91	0.363
Submucosa (μm)	40.85 ± 2.08	43.11 ± 1.05	0.206
Large intestine			
Mucosal fold height (μm)	118.99 ± 5.76	91.82 ± 10.82	0.007
Lamin propria width (μm)	13.35 ± 1.67	19.85 ± 1.88	0.004
Muscularis (μm)	74.02 ± 6.57	46.34 ± 6.40	0.007
Submucosa (μm)	28.52 ± 4.23	34.71 ± 2.06	0.085

### 3.4 The mRNA relative expression levels of nutrient transport and ion transport/channel-related genes in the small intestine

**Figure 4:**
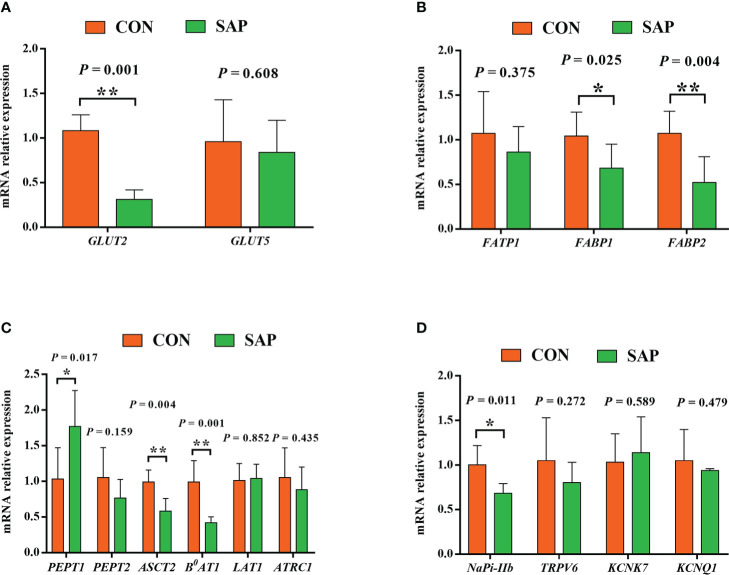
Effect of dietary soybean saponin Bb mRNA relative expression of small intestinal nutrient transporter genes **(A–C)** and ion transporters/channel genes **(D)** (*n* = 6). ^*^ *p* < 0.05; ^**^ *p* < 0.01; Student’s *t*-test. *GLUT2*, glucose transporter 2; *GLUT5*, glucose transporter 5; *FATP1*, fatty acid transporter 1; *FABP1*, fatty acid binding protein 1; *FABP2*, fatty acid binding protein 2; *PEPT1*, polypeptide transporter 1; *PEPT2*, polypeptide transporter 2; *ASCT2*, amino acid transporter 2; *B^0^AT1*, B^0,+^-type amino acid transporter 1; *ATRC1*, cationic amino acid transporter 1; *LAT1*, large neutral amino acid transporter 1; *Napi-IIb*, sodium-dependent phosphate transport protein 2b; *TRPV6*, transient receptor potential cation channel subfamily V member 6; *KCNK7*, potassium two pore domain channel subfamily K member 7; *KCNQ1*, potassium voltage-gated channel subfamily Q member 1.

As shown in **Figure 4**, the mRNA expression levels of glucose transporter *GLUT2* (A) and fatty acid transporter *FABP1* and *FABP2* (B) were markedly lower in the SAP group compared to the CON group (*p* < 0.05). The small peptide transporter *PEPT1* (C) was significantly upregulated, but the amino acid transporters *ASCT2* and *B^0^AT1* (C) were downregulated (*p* < 0.05), and the sodium–phosphorus co-transport *NaPi-IIb* (D) was also noticeably reduced (*p* < 0.05).

### 3.5 Composition and diversity of gut microbiota

**Figure 5:**
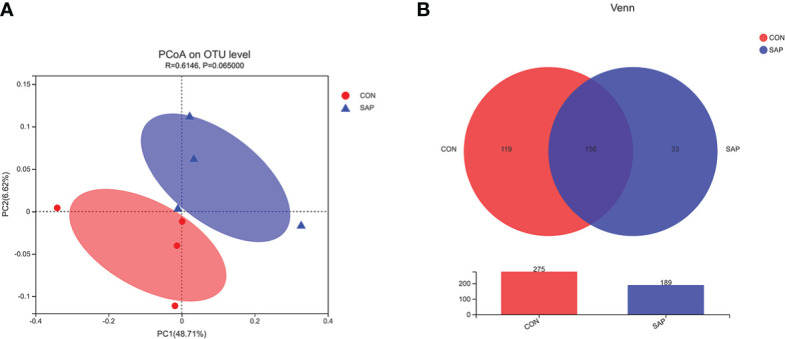
Principal coordinate analysis (PCoA) **(A)** and Venn diagram **(B)** of the intestinal bacterial community (*n* = 4). LEfSe multilevel species hierarchy tree **(A)** and LDA discriminant result table **(B)** (phylum species) (*n* = 4).

A total of 396,239 sequences and an average of 46,154.875 sequences (ranging from 40,341 to 54,541) were acquired from eight intestinal content samples in the Chinese soft-shell turtles fed CON and SAP diets. The raw data of the intestinal microbiota analysis have been uploaded to the SRA database of the NCBI with the accession PRJNA870236. The rarefaction curves of all samples tended to be stable, indicating the accuracy of the sequencing results (**Supplementary Figure S1**). The coverage index showed that the coverage of the community’s coverage reached more than 99%, which basically covered all microbial communities in the tested samples. The bacterial diversity index was assessed at the OTU level. Compared with the CON group, Shannon increased and Sobs, Simpson, Ace, and Chao decreased in the SAP group, but the differences were not significant (**Supplementary Table S3**). As shown in **Figure 5**, PCoA analysis showed that the CON and SAP groups were aggregated into two mutually disjoint groups, and Analysis of Similarities (ANSION) analysis showed that there was no significant difference between the two groups (*R* = 0.6146, *p* = 0.0650). A Venn diagram showed that the common OTUs of the CON and SAP groups were 156, and the number of unique OTUs in the CON and SAP groups were 119 and 33, respectively.

**Figure 6:**
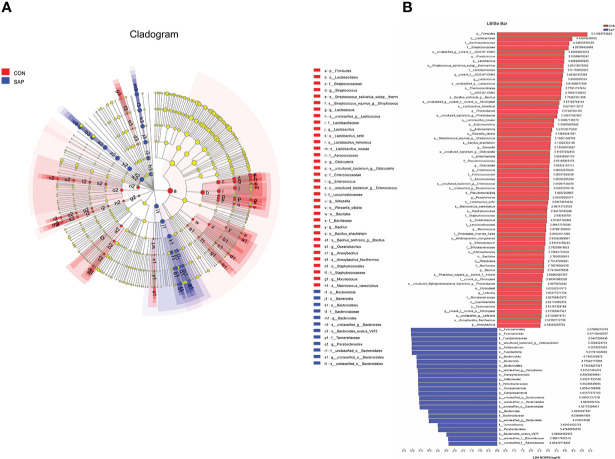
LEfSe multilevel species hierarchy tree **(A)** and LDA discriminant result table **(B)** (phylum-species) (*n* = 4). In the hierarchy tree diagram, red-colored nodes represent the microbial groups in the CON group are significantly higher than the SAP group. Blue-colored nodes represent the microbial groups in the SAP group are significantly higher than the CON group. The LDA discriminant column chart counts the microbial groups with significant effects in the two groups. The larger the LDA score is, the greater the impact of species abundance on the different effects.

LEfSe multilevel species discriminant analysis and LDA discriminant result (**Figure 6**) showed that the SAP group significantly reduced the relative richness of Firmicutes, Actinobacteriota, and Acidobacteriota and increased Fusobacteriota and Campilobacterota at phylum level. At the genus level, *Streptococcus*, *Lactobacillus*, *Lactococcus*, *Pseudomonas*, *Enterococcus*, *Macrococcus*, *Bifidobacterium*, *Bacillus*, and *Anoxybacillus* in the CON group were significantly higher than the SAP group. *Helicobacter*, *Bacteroides*, and *Parabacteroides* were the opposite trend. At the species level, compared with the CON group, the relative abundance of *Lactobacillus_rossiae*, *Bacillus_anthracis*, *Lactobacillus_helveticus*, *Bacillus_shackletonii*, *Lactobacillus_kefiri*, *Macrococcus_caseolyticus*, and *Bifidobacterium_mongoliense* were decreased and *Bacteroides_ovatus_V975* was increased in the SAP group, significantly.

### 3.6 Transcriptome analysis

The raw data of transcriptome analysis have been uploaded to the SRA database of the NCBI with the accession number PRJNA895971. A total of 319,471,138 clean reads were obtained by transcriptome analysis of six samples on the Illumina sequencing platform, and the clean reads of each sample reached more than 50,341,908, and the percentage of Q30 base was more than 93.68% (**Supplementary Table S4**). These clean reads could be mapped to the reference genome of *Pelodiscus sinensis*, respectively (**Supplementary Table S5**). The CON and SAP groups expressed a total of 14,156 genes. A total of 13,210 genes were co-expressed, and the specific expression genes were 253 and 693, respectively (**Supplementary Figure S2**).

**Figure 7:**
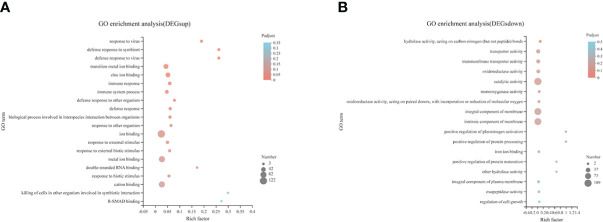
GO pathway enrichment analysis of the upregulated **(A)** and downregulated **(B)** DEGs in the comparison of CON vs. SAP.

**Figure 8:**
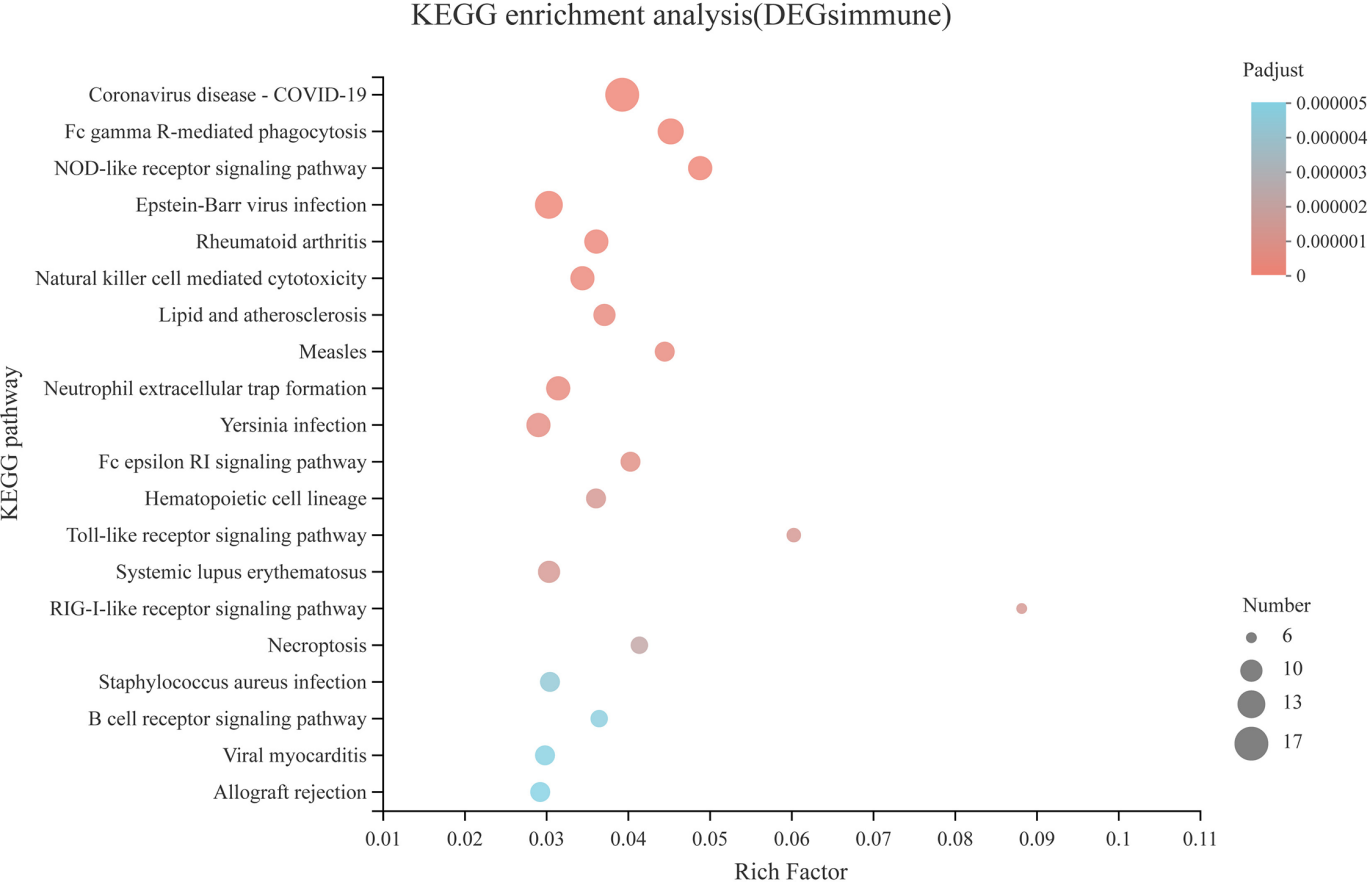
KEGG pathway enrichment analysis of the immune-related DEGs in the comparison of CON vs. SAP.

Compared with the CON diet, a total of 476 upregulated DEGs and 277 downregulated DEGs were identified in the SAP diet, respectively (**Supplementary Figure S3**). We conducted GO enrichment analysis for upregulated DEGs and downregulated DEGs, respectively. **Figures 7A, B** shows the top 20 items, respectively. We found that the SAP group significantly upregulated items that were mainly concentrated in response to the virus, ion binding, and immune response, and downregulated items that were predominantly enriched in hydrolase activity, acting on carbon–nitrogen (but not peptide) bonds, transporter activity, catalytic activity, and being an integral and intrinsic component of the membrane. We then focused our interest on the immune response. Through KEGG enrichment analysis, the genes with significantly upregulated differences were identified by “immune.” **Figure 8** shows the metabolic pathways of the top 20, and it was found that it was significantly enriched in coronavirus disease (COVID) 19, Fc gamma R-mediated phagocytosis, Nod-like receptor signaling pathway, etc.

### 3.7 QRT-PCR verification

**Figure 9:**
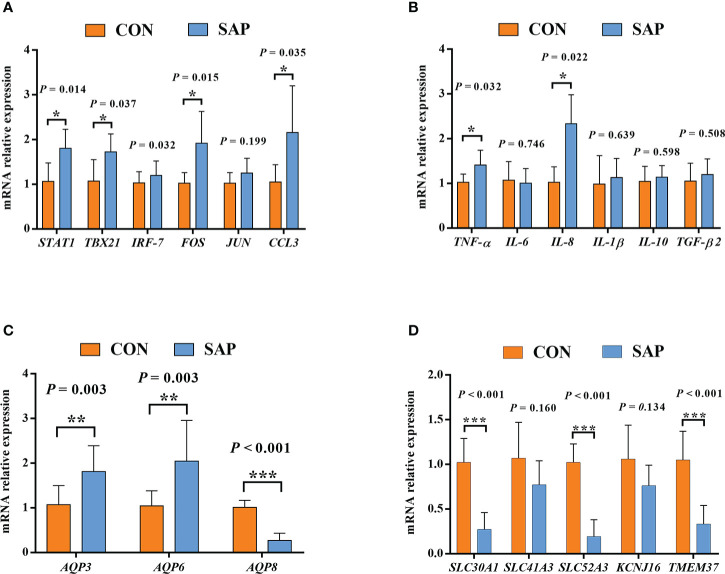
Effect of dietary soybean saponin Bb mRNA relative expression of large intestinal immune-related genes **(A, B)** and membrane transport/channels **(C, D)** (*n* = 6). ^*^ *p* < 0.05; ^**^ *p* < 0.01; ^***^ *p* < 0.001; Student’s *t*-test. *STAT1*, signal transducer and activator of transcription 1; *TBX21*, T-box transcription factor 21; *IRF7*, interferon regulatory factor 7; *FOS*, Fos proto-oncogene (AP-1 transcription factor subunit); *JUN*, Jun proto-oncogene (AP-1 transcription factor subunit); *CCL3*, C–C motif chemokine ligand 3; *TNF-α*, tumor necrosis factor alpha; *IL-6*, interleukin 6; *IL-8*, interleukin 8; *IL-1β*, interleukin 1 beta; *IL-10*, interleukin 10; *TGF-β2*, transforming growth factor beta 2; *AQP3*, aquaporin 3; *AQP6*, aquaporin 6; *AQP8*, aquaporin 8; *SLC41A3*, solute carrier family 41 member 3; *SLC30A1*, solute carrier family 30 member 1; *SLC52A3*, solute carrier family 52 member 3; *KCNJ16*, potassium inwardly rectifying channel subfamily J member 16; *TMEM37*, transmembrane protein 37.

In **Figure 9**, we selected 20 Chinese soft-shelled turtle large-intestine transcriptome differential genes for qPCR validation and found that the gene (*STAT1*, *TBX21*, *FOS*, *CCL3*, *AQP3*, *AQP3*, *AQP8*, *SLC30A1*, *SLC52A3*, *TMEM37*) validation results were consistent with the RNA-seq results with a significant difference. The genes (*IRF-7*, *JUN*, *TNF-α*, *IL-6*, *IL-8*, *IL-1β*, *IL-10*, *TGF-β2*, *SLC41A3*, *KCNJ16*) consistently matched the trend of RNA-seq results, indicating that the RNA-seq results were reliable.

## 4 Discussion

In the current study, dietary 2.46% soybean saponin Bb significantly reduced growth and feed utilization in juvenile turtles. Similarly, dietary supplementation of 5–10 g/kg soybean saponin markedly decreased the growth rate and feed efficiency in juvenile Japanese flounder (19) and zebrafish (28). Interestingly, in these two studies, the feed intake of Japanese flounder and zebrafish decreased concurrently with the addition of dietary saponin, whereas it was noticeably increased in the current study. It is speculated that the feed intake of the turtle was increased to meet body energy demands as a result of the overall decline in dietary nutrient utilization efficiency in the saponin-supplemented group. Therefore, the decreased growth performance of the turtles in the SAP group was attributed to the decrease in feed utilization and not to the feed intake. Furthermore, the decrease in feed utilization may be related to the decline of nutrient digestion and absorption, which was evidenced by the decreased nutrient digestibility coefficients.

Digestibility *in vivo* is critical to evaluating nutrient availability in feed (29). We found that 2.46% soybean saponin Bb markedly decreased the apparent digestibility coefficients (ADCs) of dry matter, crude protein, crude lipid, and gross energy in juvenile turtles. Likewise, the ADCs of crude protein or/and crude lipid were decreased by the dietary addition of soybean saponin in Japanese flounder (19) and Atlantic salmon (30). This may be explained by the fact that the poorly digested soybean saponin increased the proportion of the nonabsorbable substances in the intestine, which negatively affected the digestion and absorption of other nutrients (31). More importantly, dietary soybean saponin gradually accumulated over time in the digestive tract and progressively damaged intestinal microstructure, and then the digestive function of the alimentary system was impaired, which was also corroborated by the adverse changes of the small intestine in the current study. It is widely believed that the height of the mucosal fold in the small intestine of aquaculture animals is positively correlated with the absorption and transport efficiency of dietary nutrients (32). Additionally, the width of the lamina propria was another parameter to evaluate intestinal health, and the degree of widening of the lamina propria was negatively related to intestinal health (33). In the current study, the height of the mucosal fold was shortened in the small intestine by dietary soybean saponin, and the width of the lamina propria was increased as well. Therefore, it is implied that the digestive function of the turtle was impaired by the dietary soybean saponin.

To further investigate which nutrient transports in the small intestine were hindered by the soybean saponin, we measured the key gene expressions related with glucose, fatty acid, peptide, amino acid, phosphorus, and calcium transports, as well as relevant plasma metabolite and electrolyte concentrations. Most mammalian cells import glucose through a process of facilitated diffusion mediated by members of the membrane transporter GLUT (SLC2a) family (34). The facilitated-diffusion glucose transporter (*GLUT2*) on the basolateral surface of the enterocytes allows glucose to move from the small intestinal epithelial cells into the extracellular medium near blood capillaries (35). In this study, dietary 2.46% soybean saponin Bb significantly reduced the mRNA expression level of *GLUT2*, whereas the plasma glucose concentration was unaffected. It is probably because the plasma glucose levels in the turtle body should be regulated within a certain range through a dynamic balance mechanism to maintain body homeostasis. It is worth mentioning that the increase in feed intake in this study may be one of the strategies for maintaining blood glucose homeostasis. In terms of intestinal fatty acid absorption, the protein-mediated fatty acid uptake system is now thought to be the main pathway by which fatty acids are transported by membrane-associated fatty acid-binding proteins (FABPs) on the apical membrane of enterocytes (36). In this study, the relative expressions of *FABP1* and *FABP2* were reduced significantly by dietary soybean saponin Bb. This was in line with a previous study indicating that soy saponin significantly reduced *FABP2* expression level in the distal intestine of gilthead sea bream (11). This could also explain the decrease in turtle body lipids, apparent digestibility of crude lipid, and plasma cholesterol concentration in the soybean saponin Bb supplemented group. The terminal products of digested protein in the digestive tract of aquaculture animals were amino acids and small peptides, which were transported into/out of enterocytes *via* amino acid and di- and tripeptide transporters (37). The functional unit responsible for the absorption and transport of small peptides (dipeptides and tripeptides) in the small intestine of animals is *PEPT1*, which is located in intestinal epithelial cells (38). Dietary 2.46% soybean saponin Bb significantly increased the expression of *PEPT1* in juvenile turtles, which was consistent with the findings in zebrafish (24) and gilthead sea bream (11). This might be due to the fact that a higher proportion of dietary protein was digested in the form of small peptides in the intestine of turtles under the toxic effect of soybean saponin Bb, which could be verified backward in the results of amino acid transporter expression level (*ASCT2* and *B^0^AT1*). In addition, the transport of small peptides and free amino acids is carried out by two completely independent systems (39). In the current study, soybean saponin Bb decreased the intestinal expression of neutral amino acid transporters *ASCT2* and *B^0^AT1*, but it had no significant effect on neutral amino acid transporter *LAT1* and basic amino acid transporter *CAT1*, this may have to do with the substrate specificity of amino acid transporters. Furthermore, the absorption and utilization of dietary calcium and phosphorus are also of great importance for the growth and health of animals (40). Therefore, the key genes related to calcium (*TRPV6*, *KCNK7*, *KCNQ1*) and phosphorus (*NaPi-IIb*) transport in the intestine and relevant plasma ion concentration were also analyzed in the current study. We found that *NaPi-IIb* expression level, as well as plasma phosphorus concentration, were significantly reduced by dietary saponin Bb, whereas the intestinal calcium transport-related gene expression was not affected, despite the fact that the plasma calcium level was reduced by saponin Bb. These results implied that dietary soybean saponin Bb negatively affects calcium and phosphorus utilization in the turtle body, which might be one of the reasons for decreased growth rate in this group. The downregulated expression levels of nutrient transporters (*GLUT2*, *FABP1*, *FABP2*, *ASCT2*, *B^0^AT1*, and *NaPi-IIb*), as well as decreased plasma metabolites (cholesterol, calcium, phosphorus, and potassium) in the soybean saponin Bb group, may be attributed to impaired function of the apical membrane of enterocyte in turtles. It has been reported that the hydrophobic steroid backbone of saponins could intercalate into the hydrophobic interior of the phospholipid bilayer of enterocytes (41, 42), and then intestinal nutrient transport interfered.

It has been recognized that change in the intestinal microbial composition was closely correlated with intestinal health of the body (43, 44). On the genus level, dietary soybean saponin Bb significantly decreased the abundance of beneficial bacteria including *Lactobacillus*, *Bifidobacterium*, and *Bacillus*. It has been reported that *Lactobacillus* and *Bacillus* could promote nutrient digestion and absorption *via* secreting digestive enzymes (proteases, lipases) (45). Furthermore, *Lactobacillus* and *Bifidobacterium* exerted protective effects by counteracting enteropathogen infections (46). The decline of intestinal beneficial bacteria is also a good explanation for the decreased nutrient digestion and absorption in the saponin-supplemented group. Additionally, we found that dietary soybean saponin Bb increased the abundance of harmful bacteria with *Helicobacter* and *Bacteroides. Helicobacter* (47) and *Bacteroides* (48, 49) are the common opportunistic enteropathogens in aquatic animals, which might cause intestinal immune response and inflammation through generating endotoxins.

The dietary supplementation of soybean saponin Bb not only impaired the digestive function of the small intestine and disturbed intestinal microbiota composition but also induced inflammatory responses in the large intestine of this turtle. In the GO enrichment analysis of transcriptomics in the large intestine, the significantly upregulated genes between the treatment group and control group centered on the immune response and immune process. In order to further understand the inflammation-related metabolic pathway, we performed KEGG enrichment analysis for immune-related genes; the inflammatory response process starts from the recognition of foreign pathogenic microbial model molecules and self-injury model molecules by the cellular pattern recognition receptor PRRs to relevant transcription factors, chemokines, and cytokines. Also, the qPCR analysis proved that key transcription factors (*STAT1*, *TBX21*, *FOS*), chemokines (*CCL3*), and cytokines (*TNF*, *IL-8*) in inflammatory response were significantly upregulated in the large intestine of turtle by dietary soybean saponin Bb, which indicates that more active inflammatory responses were activated by soybean saponin Bb. In addition, in this study, soybean saponin Bb markedly upregulated the mRNA levels of *AQP3* and *AQP6* but downregulated *AQP8* in the large intestine of this turtle. Aquaporins are protein channels that promote the diffusion of water and small, uncharged molecules (such as glycerol or hydrogen peroxide) across the membrane, and they were dysregulated in response to immune and inflammatory stimulation (50). *AQP3* and *AQP8* are highly expressed in the colon, and some studies have shown that *AQP3* and *AQP8* were involved in the occurrence of intestinal inflammation (51, 52). Similarly, the intestinal *AQP8* expression level was also downregulated by the dietary addition of soybean saponin in *Sparus aurata* (11). Meanwhile, Lehmann et al. suggest that the decreased AQP8 expression is through a post-transcriptional mechanism mediated by tumor necrosis factor-α, resulting in the alterion of membrane homeostasis (53). We held the opinion that the increase of AQP3 and AQP6 may enhance the membrane permeability of H_2_0_2_, cause oxidative stress of large intestine mucosa, and lead to intestinal inflammation. However, the decrease of AQP8 is a response mechanism after inflammation, which may be a defense mechanism against oxidative stress by proinflammatory factors. The signaling pathway of water channels in intestinal inflammation needs to be further studied.

## 5 Conclusions

In a word, this study indicates that the dietary soybean saponin Bb reduced the growth performance by decreasing the digestion and absorption of macronutrients and minerals in the small intestine. Meanwhile, dietary soybean saponin Bb damaged intestinal morphology and upregulated key transcription factors, chemokines, cytokines, and aquaporins in the inflammatory response in the large intestine, leading to the intestinal inflammation of the turtle. In addition, dietary soybean saponin Bb reduced growth performance and induced intestinal inflammation, accompanied by the decreased abundance of beneficial bacteria and the increase of harmful bacteria in the digestive tract of Chinese soft-shelled turtles. This study provided a more comprehensive understanding of the adverse effects of dietary soybean saponin Bb on Chinese soft-shelled turtles, which was conducive to the development of nutritional strategies to relieve soybean meal-induced enteritis in other aquaculture animals.

## Data availability statement

The datasets presented in this study can be found in online repositories. The names of the repository/repositories and accession number(s) can be found below: https://www.ncbi.nlm.nih.gov/, PRJNA893835; https://www.ncbi.nlm.nih.gov/, PRJNA895971.

## Ethics statement

The animal study was reviewed and approved by Institutional Animal Care and Use Committee of Hebei Normal University (198013, Shijiazhuang, Hebei, China).

## Author contributions

YW: investigation, methodology, formal analysis, and writing—original draft. XJ and ZXG: investigation and data validation. LL and TL: data curation and validation. PZ: conceptualization, funding acquisition, supervision, validation, project administration, and writing—review. HL: funding acquisition, supervision, visualization, software, and writing—review. All authors contributed to the article and approved the submitted version.
