# Supplementary material for: Effect of dietary soybean saponin Bb on the growth performance, intestinal nutrient absorption, morphology, microbiota, and immune response in juvenile Chinese soft-shelled turtle (Pelodiscus sinensis)
Source: Front Immunol. 2022 Dec 23;13:1093567. doi: 10.3389/fimmu.2022.1093567 (PMC9816404; doi:10.3389/fimmu.2022.1093567)
Supplement: Supplementary file 1 [file DataSheet_1.zip › Supplementary Material 12.15.DOC]

Supplementary Material

1. Supplementary Tables

Supplementary Table S1. Primers sequence, product length, annealing temperature for genes used real time PCR

| **Genes** | **Primer Sequence (5'-3')** | **Product length (bp)** | **Annealing**  **temperature (℃)** | **Accession No.** |
| --- | --- | --- | --- | --- |
| GLUT2 | 1. GGACTTGTTCTCCTGACCACTT   R-TCCAGGCCCAACTTCAAAGA | 93 | 55 | XM_006122851.1 |
| GLUT5 | F-GAGATCGAAGAGATGCGCCA  R-GCAGAGGTTGAAGACGGACA | 72 | 62 | XM_006127095.3 |
| FATP1 | F-CTCTTTGGCCTCTCGGTGTT  R-AGGGCCACTTTGTTAGGGTG | 122 | 60 | XM_025189298.1 |
| FABP1 | F-GGAGAGAAGGTCAAGGCTGTT  R-TCGTGTAGCTGATGTCTCCT | 139 | 60 | XM_006111611.3 |
| FABP2 | F-GAAAGCTAGGAGCTCACGACA  R-CCAGTGTCCAATTTCCATTGAGT | 171 | 60 | XM_006112602.3 |
| PEPT1 | F-CAATACAGTTCATATGGCTT  R-TAGCACAGATTTCATATTGGA | 127 | 52 | XM_006128130.3 |
| PEPT2 | F-GGAGCAAATTCTTTTCCGTCT  R-ACATCCCCTCTCAATACAGG | 88 | 56 | XM_006137687.3 |
| ASCT2 | F-AGGACTGTGGGCGATTCTTG  R-TCCATGGGTCACAATCACGG | 80 | 60 | XM_006125862.3 |
| B^0^AT1 | 1. ATGGTGTTGAAGGAACTGGCTTGG   R-GAGAATGGACCACAAAGGCGAGAC | 86 | 60 | XM_006137063.3 |
| ATRCI | F-GTCGCTTACTTTGGCGTGTC  R-CATAGTTGGCACCCTCCCAG | 118 | 62 | XM_014579335.1 |
| LAT1 | F-CCTGCTTATTCCTGATCGTGGTCTC  R-ATCCCGCTGAGAATGATGGCAAAG | 82 | 60 | XM_025182424.1 |
| NaPi-IIb | F-TGTCAAACCCTGTTGCTGGT  R-AGGTACCAATGTTTGCCCCC | 159 | 55 | XM_025179010.1 |
| TRPV6 | F-CGTCCCTATTTCTGTATCCT  R-AGTGTTGTCCCTTTCGTG | 123 | 58 | XM_025186839.1 |
| KCNK7 | F-CCTTATCCGATTCTTTGCTC  R-CTCCTTGTCTGGTTGGCTC | 145 | 55 | XM_014577797.2 |
| KCNQ1 | F-TCGGGTCTCCATCTACAGCA  R-CCGGTGGGTCTCTCTAGGAA | 90 | 62 | XM_006134905.3 |
| STAT1 | F-GATGGCCTTATTCCTTGGAC  R-GACCTTTAACAAAGCACGTTC | 180 | 57 | XM_014570875.2 |
| TBX21 | F-ACGTGACGGAGGTGAAGGATGG  R-GAACTGCGTCTCTGGGAAGATGAAG | 86 | 64 | XM_014574113.1 |
| IRF7 | F-ATCTCGCCAAGTGGAAAACCAA  R-ACCTTTGAGTTATCCTGCACCA | 82 | 58 | XM_006124885.3 |
| CCL3 | F-GTGTTTACCACGAGGCAGGG  R-CGACAGGCTGGTAACGTACTC | 81 | 64 | XM_006115865.3 |
| JUN | F-AATCAGAATACGATGCCCAG  R-GTGCGCTAGGGTTAAAGTTG | 157 | 65 | XM_006133503.1 |
| FOS | F-TGCCAACTTCGTACCCACA  R-TCCAATGCTTTGCCCTCT | 196 | 60 | XM_006117769.3 |
| SLC41A3 | F-AATTGTGCGACTGATGTGGAGGAAG  R-GAGCCAAACCAAACGGAAGCAAAG | 124 | 60 | XM_006131255.3 |
| SLC30A1 | F-CTGTCTTCCTCACCGCCCTC  R-TCCCACTCCAATCACCACAA | 104 | 60 | XM_014573963.2 |
| SLC52A3 | F-CCTAGCAACATCAGCCTGCACT  R-TAGCGAGTTCACCCAGGCCACA | 190 | 64 | XM_025179620.1 |
| KCNJ16 | F-CTTTCTTGGCTGTTCTTCGG  R-CACAACGAAAACCATAACCT | 169 | 62 | XM_014576576.2 |
| TMEM37 | F-TGCTCACAGAAGACCCAAGAAATCG  R-TTGATGACAGGACCACTGCTATTGC | 92 | 62 | XM_006137629.1 |
| AQP3 | F-CCAGTTCATCGGCACAGCATCC  R-CCCAATCACCAGGACGACAAAGC | 121 | 63 | XM_006122997.3 |
| AQP6 | F-AATGTGGAAGGAAGTGCTGTCTGTG  R-AGAAGACGTAGATGGAGGTGGCTAG | 80 | 63 | XM_025188749.1 |
| AQP8 | F-ATCAAGGAGGTGGAAATGGA  R-ACCCGATGAAGATGAACAGC | 118 | 60 | XM_006133833.3 |
| TNF-α | F-GCCCATGTCGTAGCTTCCCA  R-TCCACCAGCTTCATCCCGTTC | 92 | 65 | XM_014575959.2 |
| IL-8 | F-AGCACGCACGCTAAGTTCATCC  R-TGATTTCGACGTTCTGGCAGTGAG | 88 | 65 | XM_006125396.3 |
| IL-1β | F-AGCTGAAGTACACGGAGAAGACCTC  R-GGCGTCCAAGATGCTGCTCAAG | 134 | 65 | KC430862.1 |
| IL-10 | F-AAAGCAATCAACAGCAGCAAAGACG  R-AGTGTCTTCCTGAGGTCCAGCAG | 80 | 65 | KT203380.1 |
| TGF-β2 | F-CGAAAATGCCATCCCACCA  R-TGCCTTTGAATTCTGCAAACGA | 136 | 61 | XM_006115568.1 |
| RPS18 | F-CCTTCGCCATCACCGCTATCAAG  R-GGTCAGGTCAATGTCGGCTTTCC | 80 | 58 | XM_006130627.3 |
| ACTB | F-AATCGTGCGTGACATCAAGGAGAAG  R-CCTGAACCTCTCGTTGCCAATGG | 148 | 58 | XM_006112915.3 |

GLUT2: Glucose transporter 2; GLUT5: Glucose transporter 5; FATP1: Fatty acid transporter 1; FABP1: Fatty acid binding protein1; FABP2: Fatty acid binding protein 2; PEPT1: Polypeptide transporter 1; PEPT2: Polypeptide transporter 2; ASCT2(SLC1A5): Amino acid transporter 2 ; B^0^AT1(SLC6A19): B^0,+^-type amino acid transporter 1; ATRC1(SLC7A1): Cationic amino acid transporter 1; LAT1(SLC7A5): Large neutral amino acid transporter 1; Napi-IIb: Sodium-dependent phosphate transport protein 2b; TRPV6: Tran-sient receptor potential cation channel subfamily V member 6; KCNK7: Potassium two pore domain channel subfamily K member 7; KCNQ1: Potassium voltage-gated channel subfamily Q member 1; STAT1: signal transducer and activator of transcription 1; TBX21: T-box transcription factor 21; IRF7: Interferon regulatory factor 7; CCL3: C-C motif chemokine ligand 3; JUN: Jun proto-oncogene, AP-1 transcription factor subunit; FOS: Fos proto-oncogene, AP-1 transcription factor subunit; SLC41A3:Solute carrier family 41 member 3; SLC30A1: Solute carrier family 30 member 1; SLC52A3: Solute carrier family 52 member 3; KCNJ16: Potassium inwardly rectifying channel subfamily J member 16; TMEM37: Transmembrane protein 37; AQP3: Aquaporin 3; AQP6: Aquaporin 3; AQP8: Aquaporin 8; TNF-α: [Tumor necrosis factor](https://www.ncbi.nlm.nih.gov/gene/7124) alpha; IL-6: Interleukin 6; IL-8:Interleukin 8; IL-1β: interleukin 1 beta; IL-10: Interleukin 10; TGF-β2: Transforming growth factor beta 2; RPS18: Ribosomal protein S18; ACTB: β-actin

**Supplementary Table S2. Formulas of growth index, body index and nutrient apparent digestibility**

| Indices | Formulas |
| --- | --- |
| SR (%) | Survival rate (%) = (final turtle number / initial turtle number) × 100 |
| FR (%/d) | Feeding rate (% BW/d) = 100 × dry feed intake / [days × (FBW + IBW) / 2] |
| WGR (%) | Weight gain rate (%) = 100 × (FBW - IBW) / IBW |
| SGR (%/d) | Specific growth rate (%/d) = 100 × [Ln (FBW) – Ln (IBW] / days |
| FCR | Feed conversion ratio = dry feed intake / fresh body weight gain |
| PER | Protein efficiency ratio = fresh body weight gain / protein intake |
| PDR (%) | Protein deposition ratio (%) = protein gain/protein intake × 100 |
| FDR (%) | Fat deposition ratio (%) = fat gain/protein intake × 100 |
| VSI (%) | Viscerosomatic index (%) =100×Visceral mass weight /body weight |
| HSI (%) | Hepatosomatic index (%) =100×liver weight/body weight |
| FSI (%) | Fatsomatic index (%) = 100× fat weight/body weight |
| ADC_DM_ | Apparent digestibility coefficient of dry matter (ADC_DM_, %) = 100 × (1 - D_Y_ / F_Y_) |
| ADC_CP_ | Apparent digestibility coefficient of crude protein (ADC_CP_, %) = 100 × [1- (F_CP_ / D_CP_) × (D_Y_ / F_Y_)] |
| ADC_CL_ | Apparent digestibility coefficient of crude lipid (ADC_CL_, %) = 100 × [1- (F_CL_ / D_CL_) × (D_Y_ / F_Y_)] |
| ADC_GE_ | Apparent digestibility coefficient of gross energy (ADC_GE_, %) = 100 × [1- (F_GE_ / D_GE_) × (D_Y_ / F_Y_)] |

IBW: Initial body weight(g); FBW: Final body weight (g);D_Y_, Dietary yttrium content (%); F_Y_, Fecal yttrium content (%); D_CP_, Dietary crude protein content (%); F_CP_, Fecal crude protein content (%); D_CF_, Dietary crude lipid content (%); F_CF_, Fecal crude lipid content (%); D_GE_, Gross energy (KJ / g); F_GE_, Fecal gross energy (KJ / g).

**Supplementary Table S3. Alpha diversity indexes based on 16 s rDNA gene sequence (n = 4).**

| Parameters | CON | SAP | *P-*value |
| --- | --- | --- | --- |
| Sobs | 142 ± 50.93 | 115.25 ± 14.98 | 0.353 |
| Shannon | 2.15 ± 0.74 | 2.23 ± 0.56 | 0.869 |
| Simpson | 0.26 ± 0.14 | 0.22 ± 0.12 | 0.684 |
| Ace | 171.43 ± 36.06 | 138.43 ± 6.84 | 0.122 |
| Chao | 167.89 ± 37.16 | 136.56 ± 8.51 | 0.151 |
| Coverage | 1.00 ± 0.00 | 1.00 ± 0.00 | 0.193 |

**Supplementary Table S4. Large intestine transcriptome sequencing data**

| Sample | Raw reads | Raw bases | Clean reads | Clean bases | Error rate (%) | Q20(%) | Q30(%) | GC content (%) |
| --- | --- | --- | --- | --- | --- | --- | --- | --- |
| SAP | 51938814 | 7842760914 | 50341908 | 7351351874 | 0.0249 | 97.98 | 94.29 | 50.57 |
| SAP | 55702306 | 8411048206 | 53805796 | 7733978305 | 0.0248 | 98.01 | 94.39 | 48.72 |
| SAP | 55648064 | 8402857664 | 53482814 | 7817081724 | 0.0254 | 97.8 | 93.88 | 51.12 |
| CON | 57893900 | 8741978900 | 55770238 | 8172310357 | 0.0256 | 97.74 | 93.68 | 49.18 |
| CON | 53288852 | 8046616652 | 51741992 | 7560277109 | 0.0252 | 97.89 | 94.02 | 50.07 |
| CON | 56439210 | 8522320710 | 54328390 | 7930921301 | 0.0255 | 97.77 | 93.78 | 49.54 |

**Supplementary Table S5. Large intestine transcriptome comparison of data**

| Sample | Total reads | Total mapped | Multiple mapped | Uniquely mapped |
| --- | --- | --- | --- | --- |
| SAP | 50341908 | 39543397 (78.55%) | 1681743 (3.34%) | 37861654 (75.21%) |
| SAP | 53805796 | 43574172 (80.98%) | 1704185 (3.17%) | 41869987 (77.82%) |
| SAP | 53482814 | 41238813 (77.11%) | 1769731 (3.31%) | 39469082 (73.8%) |
| CON | 55770238 | 44521566 (79.83%) | 1860016 (3.34%) | 42661550 (76.5%) |
| CON | 51741992 | 41223434 (79.67%) | 1787988 (3.46%) | 39435446 (76.22%) |
| CON | 54328390 | 43373472 (79.84%) | 1842385 (3.39%) | 41531087 (76.44%) |

**Supplementary Table S6. Full name of the abbreviation**

| Abbreviations | Full name |
| --- | --- |
| ADCs | Apparent digestibility coefficients |
| ANSION | Analysis of Similarities |
| *AQP3* | Aquaporin 3 |
| *AQP6* | Aquaporin 6 |
| *AQP8* | Aquaporin 8 |
| *ASCT2* | Amino acid transporter 2 |
| *ATRC1* | Cationic amino acid transporter 1 |
| *B^0^AT1* | B^0,+^-type amino acid transporter 1 |
| C3 | Complement 3 |
| *CCL3* | C-C motif chemokine ligand 3 |
| *FABP1* | Fatty acid binding protein 1 |
| *FABP2* | Fatty acid binding protein 2 |
| *FATP1* | Fatty acid transporter 1 |
| FBW | Final body weight |
| FCR | Feed conversion ratio |
| FDR | Fat deposition ratio |
| FOS | Fos proto-oncogene, AP-1 transcription factor subunit |
| FR | Feeding rate |
| FSI | Fatsomatic index |
| *GLUT2* | Glucose transporter 2 |
| *GLUT5* | Glucose transporter 5 |
| GO | Gene ontology |
| HSI | Hepatosomatic index |
| *IL-10* | Interleukin 10 |
| *IL-1β* | Interleukin 1 beta |
| *IL-6* | Interleukin 6 |
| *IL-8* | Interleukin 8 |
| *IL-8* | Interleukin 8 |
| *IRF7* | Interferon regulatory factor 7 |
| *JUN* | Jun proto-oncogene, AP-1 transcription factor subunit |
| *KCNJ16* | Potassium two pore domain channel subfamily K member 7 |
| *KCNK7* | Potassium two pore domain channel subfamily K member 7 |
| *KCNQ1* | Potassium voltage-gated channel subfamily Q member 1 |
| *KEGG* | Kyoto encyclopedia of genes and genomes |
| *LAT1* | Large neutral amino acid transporter 1 |
| LDA | Linear Discriminant Analysis |
| LEfSE | Linear Discriminant Analysis Effect Size |
| *NaPi-IIb* | Sodium-dependent phosphate transport protein 2b |
| NCBI | National Center for Biotechnology Information |
| PDR | Protein deposition ratio |
| *PEPT1* | Polypeptide transporter 1 |
| *PEPT2* | Polypeptide transporter 2 |
| PER | Protein efficiency ratio |
| qPCR | Real-time quantitative polymerase chain reaction |
| RPS18 | Ribosomal protein S18 |
| SAR | Sequence read archive |
| SGR | Specific growth rate |
| *SLC30A1* | Solute carrier family 30 member 1 |
| *SLC41A3* | Solute carrier family 41 member 3 |
| *SLC52A3* | Solute carrier family 52 member 3 |
| SR | Survival rate |
| *STAT1* | Signal transducer and activator of transcription 1 |
| *TBX21* | T-box transcription factor 21 |
| *TGF-β2* | Transforming growth factor beta 2 |
| *TMEM37* | Transmembrane protein 37 |
| *TNF-α* | [Tumor necrosis factor](https://www.ncbi.nlm.nih.gov/gene/7124) alpha |
| *TRPV6* | Transient receptor potential cation channel subfamily V member 6 |
| VSI | Viscerosomatic index |
| WGR | Weight gain rate |
| Y_2_O_3_ | Yttrium trioxide |

## Supplementary Figures


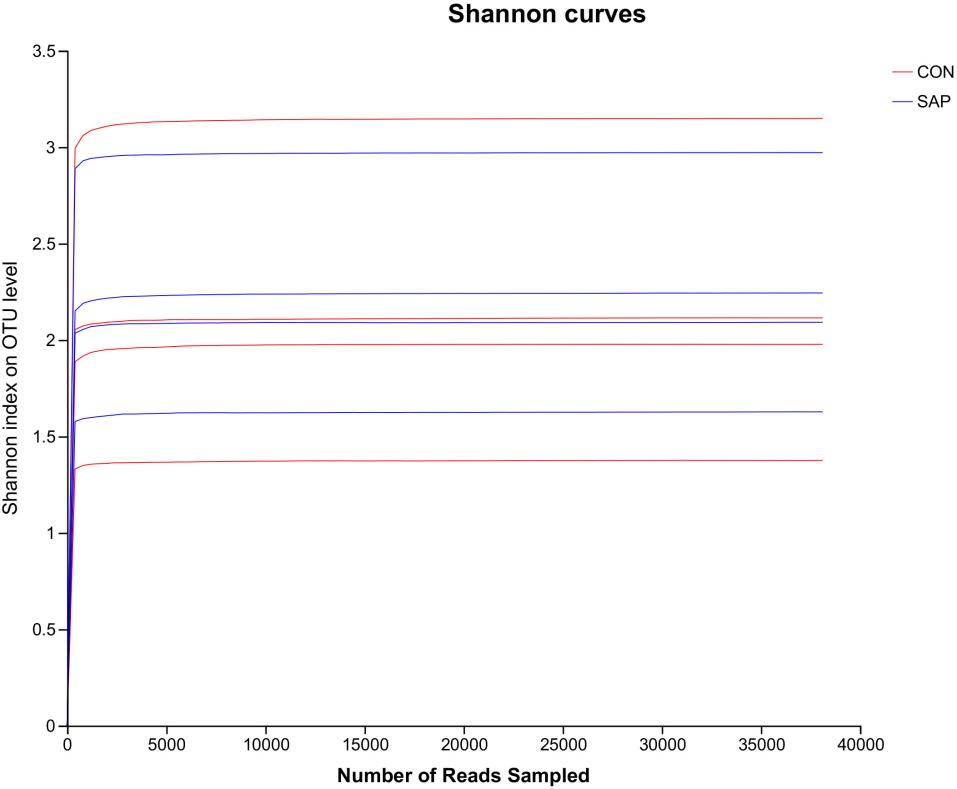


**Supplementary Figure S1. Rarefaction curves of intestinal microbiota（n=4）**

**
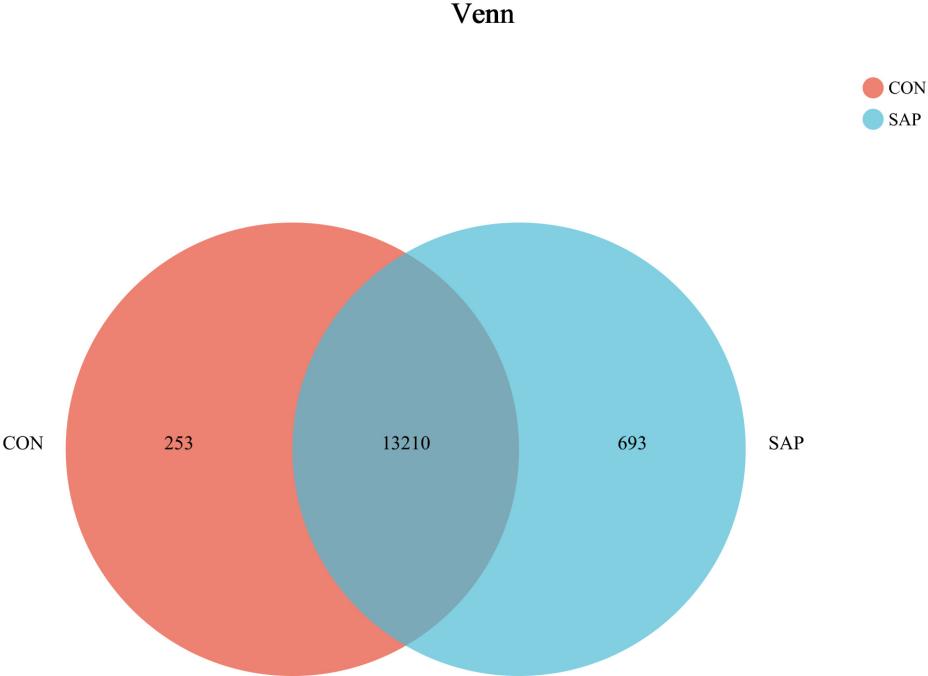
**

**Supplementary Figure S2. Venn diagram of transcriptome expressed genes (n=3)**


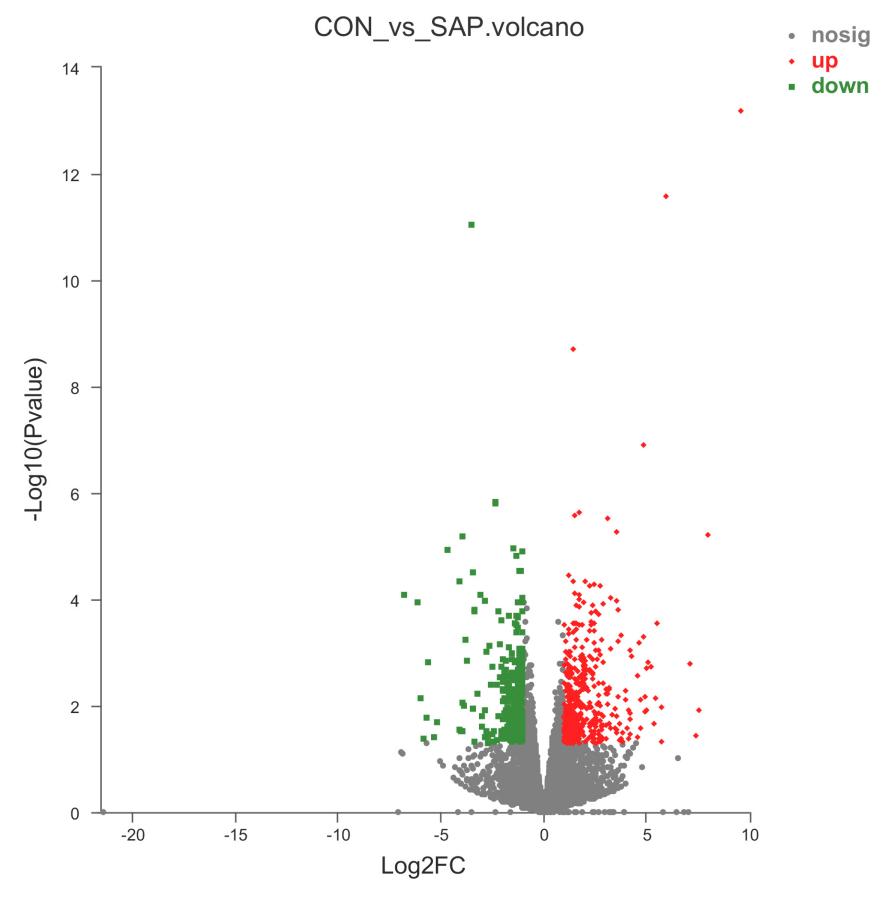


**Supplementary Figure S3. Volcano Plot of DEGs (n=3)**
