# Supplementary material for: Effect of dietary soybean saponin Bb on the growth performance, intestinal nutrient absorption, morphology, microbiota, and immune response in juvenile Chinese soft-shelled turtle (Pelodiscus sinensis)
Source: Front Immunol. 2022 Dec 23;13:1093567. doi: 10.3389/fimmu.2022.1093567 (PMC9816404; doi:10.3389/fimmu.2022.1093567)
Supplement: Supplementary file 1 [file DataSheet_1.zip › Supplementary Material 12.15.pdf]

*Supplementary Material*

## 1. Supplementary Tables

**Supplementary Table S1. Primers sequence, product length, annealing temperature for genes used real time PCR**

| Genes | Primer Sequence (5'-3') | Product length (bp) | Annealing temperature (℃) | Accession No. |
|---|---|---|---|---|
| GLUT2 | F-GGACTTGTTCTCCTGACCACTT | 93 | 55 | XM_006122851.1 |
| | R-TCCAGGCCCAACTTCAAAGA | | | |
| GLUT5 | F-GAGATCGAAGAGATGCGCCA | 72 | 62 | XM_006127095.3 |
| | R-GCAGAGGTTGAAGACGGACA | | | |
| FATP1 | F-CTCTTTGGCCTCTCGGTGTT | 122 | 60 | XM_025189298.1 |
| | R-AGGGCCACTTTGTTAGGGTG | | | |
| FABP1 | F-GGAGAGAAGGTCAAGGCTGTT | 139 | 60 | XM_006111611.3 |
| | R-TCGTGTAGCTGATGTCTCCT | | | |
| FABP2 | F-GAAAGCTAGGAGCTCACGACA | 171 | 60 | XM_006112602.3 |
| | R-CCAGTGTCCAATTTCCATTGAGT | | | |
| PEPT1 | F-CAATACAGTTCATATGGCTT | 127 | 52 | XM_006128130.3 |
| | R-TAGCACAGATTTCATATTGGA | | | |
| PEPT2 | F-GGAGCAAATTCTTTTCCGTCT | 88 | 56 | XM_006137687.3 |
| | R-ACATCCCCTCTCAATACAGG | | | |
| ASCT2 | F-AGGACTGTGGGCGATTCTTG | 80 | 60 | XM_006125862.3 |

| | | | | |
|---|---|---|---|---|
| | R-TCCATGGGTCACAATCACGG | | | |
| $B^0AT1$ | F-ATGGTGTTGAAGGAACTGGCTTGG | 86 | 60 | XM_006137063.3 |
| | R-GAGAATGGACCACAAAGGCGAGAC | | | |
| ATRCI | F-GTCGCTTACTTTGGCGTGTC | 118 | 62 | XM_014579335.1 |
| | R-CATAGTTGGCACCCTCCCAG | | | |
| LAT1 | F-CCTGCTTATTCCTGATCGTGGTCTC | 82 | 60 | XM_025182424.1 |
| | R-ATCCCGCTGAGAATGATGGCAAAG | | | |
| NaPi-IIb | F-TGTCAAACCCTGTTGCTGGT | 159 | 55 | XM_025179010.1 |
| | R-AGGTACCAATGTTTGCCCCC | | | |
| TRPV6 | F-CGTCCCTATTTCTGTATCCT | 123 | 58 | XM_025186839.1 |
| | R-AGTGTTGTCCCTTTCGTG | | | |
| KCNK7 | F-CCTTATCCGATTCTTTGCTC | 145 | 55 | XM_014577797.2 |
| | R-CTCCTTGTCTGGTTGGCTC | | | |
| KCNQ1 | F-TCGGGTCTCCATCTACAGCA | 90 | 62 | XM_006134905.3 |
| | R-CCGGTGGGTCTCTCTAGGAA | | | |
| STAT1 | F-GATGGCCTTATTCCTTGGAC | 180 | 57 | XM_014570875.2 |
| | R-GACCTTTAACAAAGCACGTTC | | | |
| TBX21 | F-ACGTGACGGAGGTGAAGGATGG | 86 | 64 | XM_014574113.1 |
| | R-GAACTGCGTCTCTGGGAAGATGAAG | | | |
| IRF7 | F-ATCTCGCCAAGTGGAAAACCAA | 82 | 58 | XM_006124885.3 |
| | R-ACCTTTGAGTTATCCTGCACCA | | | |
| CCL3 | F-GTGTTTACCACGAGGCAGGG | 81 | 64 | XM_006115865.3 |

| | | | | |
|---|---|---|---|---|
| | R-CGACAGGCTGGTAACGTACTC | | | |
| JUN | F-AATCAGAATACGATGCCCAG | 157 | 65 | XM_006133503.1 |
| | R-GTGCGCTAGGGTTAAAGTTG | | | |
| FOS | F-TGCCAACTTCGTACCCACA | 196 | 60 | XM_006117769.3 |
| | R-TCCAATGCTTTGCCCTCT | | | |
| SLC41A3 | F-AATTGTGCGACTGATGTGGAGGAAG | 124 | 60 | XM_006131255.3 |
| | R-GAGCCAAACCAAACGGAAGCAAAG | | | |
| SLC30A1 | F-CTGTCTTCCTCACCGCCCTC | 104 | 60 | XM_014573963.2 |
| | R-TCCCACTCCAATCACCACAA | | | |
| SLC52A3 | F-CCTAGCAACATCAGCCTGCACT | 190 | 64 | XM_025179620.1 |
| | R-TAGCGAGTTCACCCAGGCCACA | | | |
| KCNJ16 | F-CTTTCTTGGCTGTTCTTCGG | 169 | 62 | XM_014576576.2 |
| | R-CACAACGAAAACCATAACCT | | | |
| TMEM37 | F-TGCTCACAGAAGACCCAAGAAATCG | 92 | 62 | XM_006137629.1 |
| | R-TTGATGACAGGACCACTGCTATTGC | | | |
| AQP3 | F-CCAGTTCATCGGCACAGCATCC | 121 | 63 | XM_006122997.3 |
| | R-CCCAATCACCAGGACGACAAAGC | | | |
| AQP6 | F-AATGTGGAAGGAAGTGCTGTCTGTG | 80 | 63 | XM_025188749.1 |
| | R-AGAAGACGTAGATGGAGGTGGCTAG | | | |
| AQP8 | F-ATCAAGGAGGTGGAAATGGA | 118 | 60 | XM_006133833.3 |
| | R-ACCCGATGAAGATGAACAGC | | | |
| TNF-α | F-GCCCATGTCGTAGCTTCCCA | 92 | 65 | XM_014575959.2 |
| | R-TCCACCAGCTTCATCCCGTTC | | | |

| | | | | |
|---|---|---|---|---|
| IL-8 | F-AGCACGCACGCTAAGTTCATCC<br>R-TGATTTCGACGTTCTGGCAGTGAG | 88 | 65 | XM_006125396.3 |
| IL-1β | F-AGCTGAAGTACACGGAGAAGACCTC<br>R-GGCGTCCAAGATGCTGCTCAAG | 134 | 65 | KC430862.1 |
| IL-10 | F-AAAGCAATCAACAGCAGCAAAGACG<br>R-AGTGTCTTCCTGAGGTCCAGCAG | 80 | 65 | KT203380.1 |
| TGF-β2 | F-CGAAAATGCCATCCCACCA<br>R-TGCCTTTGAATTCTGCAAACGA | 136 | 61 | XM_006115568.1 |
| RPS18 | F-CCTTCGCCATCACCGCTATCAAG<br>R-GGTCAGGTCAATGTCGGCTTTCC | 80 | 58 | XM_006130627.3 |
| ACTB | F-AATCGTGCGTGACATCAAGGAGAAG<br>R-CCTGAACCTCTCGTTGCCAATGG | 148 | 58 | XM_006112915.3 |

GLUT2: Glucose transporter 2; GLUT5: Glucose transporter 5; FATP1: Fatty acid transporter 1; FABP1: Fatty acid binding protein1; FABP2: Fatty acid binding protein 2; PEPT1: Polypeptide transporter 1; PEPT2: Polypeptide transporter 2; ASCT2(SLC1A5): Amino acid transporter 2 ; $B^0$AT1(SLC6A19): $B^{0,+}$-type amino acid transporter 1; ATRC1(SLC7A1): Cationic amino acid transporter 1; LAT1(SLC7A5): Large neutral amino acid transporter 1; Napi-IIb: Sodium-dependent phosphate transport protein 2b; TRPV6: Tran-sient receptor potential cation channel subfamily V member 6; KCNK7: Potassium two pore domain channel subfamily K member 7; KCNQ1: Potassium voltage-gated channel subfamily Q member 1; STAT1: signal transducer and activator of transcription 1; TBX21: T-box transcription factor 21; IRF7: Interferon regulatory factor 7; CCL3: C-C motif chemokine ligand 3; JUN: Jun proto-oncogene, AP-1 transcription factor subunit; FOS: Fos proto-oncogene, AP-1 transcription factor subunit; SLC41A3:Solute carrier family 41 member 3; SLC30A1: Solute carrier family 30 member 1; SLC52A3: Solute carrier family 52 member 3; KCNJ16: Potassium inwardly rectifying channel subfamily J member 16; TMEM37: Transmembrane protein 37; AQP3: Aquaporin 3; AQP6: Aquaporin 3; AQP8: Aquaporin 8; TNF-α: Tumor necrosis factor alpha; IL-6: Interleukin 6; IL-8:Interleukin 8; IL-1β: interleukin 1 beta; IL-10: Interleukin 10; TGF-β2: Transforming growth factor beta 2; RPS18: Ribosomal protein S18; ACTB: β-actin

**Supplementary Table S2. Formulas of growth index, body index and nutrient apparent digestibility**

| Indices | Formulas |
|---|---|
| SR (%) | Survival rate (%) = (final turtle number / initial turtle number) × 100 |
| FR (%/d) | Feeding rate (% BW/d) = 100 × dry feed intake / [days × (FBW + IBW) / 2] |
| WGR (%) | Weight gain rate (%) = 100 × (FBW - IBW) / IBW |
| SGR (%/d) | Specific growth rate (%/d) = 100 × [Ln (FBW) – Ln (IBW] / days |
| FCR | Feed conversion ratio = dry feed intake / fresh body weight gain |
| PER | Protein efficiency ratio = fresh body weight gain / protein intake |
| PDR (%) | Protein deposition ratio (%) = protein gain/protein intake × 100 |
| FDR (%) | Fat deposition ratio (%) = fat gain/protein intake × 100 |
| VSI (%) | Viscerosomatic index (%) =100×Visceral mass weight /body weight |
| HSI (%) | Hepatosomatic index (%) =100×liver weight/body weight |
| FSI (%) | Fatsomatic index (%) = 100× fat weight/body weight |
| $ADC_{DM}$ | Apparent digestibility coefficient of dry matter ($ADC_{DM}$, %) = 100 × (1 - $D_Y$ / $F_Y$) |
| $ADC_{CP}$ | Apparent digestibility coefficient of crude protein ($ADC_{CP}$, %) = 100 × [1- ($F_{CP}$ / $D_{CP}$) × ($D_Y$ / $F_Y$)] |
| $ADC_{CL}$ | Apparent digestibility coefficient of crude lipid ($ADC_{CL}$, %) = 100 × [1- ($F_{CL}$ / $D_{CL}$) × ($D_Y$ / $F_Y$)] |
| $ADC_{GE}$ | Apparent digestibility coefficient of gross energy ($ADC_{GE}$, %) = 100 × [1- ($F_{GE}$ / $D_{GE}$) × ($D_Y$ / $F_Y$)] |

IBW: Initial body weight(g); FBW: Final body weight (g);$D_Y$, Dietary yttrium content (%); $F_Y$, Fecal yttrium content (%); $D_{CP}$, Dietary crude protein content (%); $F_{CP}$, Fecal crude protein content (%);

$D_{CF}$, Dietary crude lipid content (%); $F_{CF}$, Fecal crude lipid content (%); $D_{GE}$, Gross energy (KJ / g); $F_{GE}$, Fecal gross energy (KJ / g).

**Supplementary Table S3. Alpha diversity indexes based on 16 s rDNA gene sequence (n = 4).**

| Parameters | CON | SAP | *P*-value |
|---|---|---|---|
| Sobs | 142 ± 50.93 | 115.25 ± 14.98 | 0.353 |
| Shannon | 2.15 ± 0.74 | 2.23 ± 0.56 | 0.869 |
| Simpson | 0.26 ± 0.14 | 0.22 ± 0.12 | 0.684 |
| Ace | 171.43 ± 36.06 | 138.43 ± 6.84 | 0.122 |
| Chao | 167.89 ± 37.16 | 136.56 ± 8.51 | 0.151 |
| Coverage | 1.00 ± 0.00 | 1.00 ± 0.00 | 0.193 |

**Supplementary Table S4. Large intestine transcriptome sequencing data**

| Sample | Raw reads | Raw bases | Clean reads | Clean bases | Error rate (%) | Q20(%) | Q30(%) | GC content (%) |
|---|---|---|---|---|---|---|---|---|
| SAP | 51938814 | 7842760914 | 50341908 | 7351351874 | 0.0249 | 97.98 | 94.29 | 50.57 |
| SAP | 55702306 | 8411048206 | 53805796 | 7733978305 | 0.0248 | 98.01 | 94.39 | 48.72 |
| SAP | 55648064 | 8402857664 | 53482814 | 7817081724 | 0.0254 | 97.8 | 93.88 | 51.12 |
| CON | 57893900 | 8741978900 | 55770238 | 8172310357 | 0.0256 | 97.74 | 93.68 | 49.18 |
| CON | 53288852 | 8046616652 | 51741992 | 7560277109 | 0.0252 | 97.89 | 94.02 | 50.07 |
| CON | 56439210 | 8522320710 | 54328390 | 7930921301 | 0.0255 | 97.77 | 93.78 | 49.54 |

**Supplementary Table S5. Large intestine transcriptome comparison of data**

| Sample | Total reads | Total mapped | Multiple mapped | Uniquely mapped |
|---|---|---|---|---|
| SAP | 50341908 | 39543397 (78.55%) | 1681743 (3.34%) | 37861654 (75.21%) |
| SAP | 53805796 | 43574172 (80.98%) | 1704185 (3.17%) | 41869987 (77.82%) |
| SAP | 53482814 | 41238813 (77.11%) | 1769731 (3.31%) | 39469082 (73.8%) |
| CON | 55770238 | 44521566 (79.83%) | 1860016 (3.34%) | 42661550 (76.5%) |
| CON | 51741992 | 41223434 (79.67%) | 1787988 (3.46%) | 39435446 (76.22%) |
| CON | 54328390 | 43373472 (79.84%) | 1842385 (3.39%) | 41531087 (76.44%) |

**Supplementary Table S6. Full name of the abbreviation**

| Abbreviations | Full name |
|---|---|
| ADCs | Apparent digestibility coefficients |
| ANSION | Analysis of Similarities |
| *AQP3* | Aquaporin 3 |
| *AQP6* | Aquaporin 6 |
| *AQP8* | Aquaporin 8 |
| *ASCT2* | Amino acid transporter 2 |
| *ATRC1* | Cationic amino acid transporter 1 |
| *$B^0AT1$* | $B^{0,+}$-type amino acid transporter 1 |
| C3 | Complement 3 |

| | |
|---|---|
| *CCL3* | C-C motif chemokine ligand 3 |
| *FABP1* | Fatty acid binding protein 1 |
| *FABP2* | Fatty acid binding protein 2 |
| *FATP1* | Fatty acid transporter 1 |
| FBW | 1.1.1 Final body weight |
| FCR | Feed conversion ratio |
| FDR | Fat deposition ratio |
| FOS | Fos proto-oncogene, AP-1 transcription factor subunit |
| FR | Feeding rate |
| FSI | Fatsomatic index |
| *GLUT2* | Glucose transporter 2 |
| *GLUT5* | Glucose transporter 5 |
| GO | Gene ontology |
| HSI | Hepatosomatic index |
| *IL-10* | Interleukin 10 |
| *IL-1β* | Interleukin 1 beta |
| *IL-6* | Interleukin 6 |
| *IL-8* | Interleukin 8 |

| | |
|---|---|
| *IL-8* | Interleukin 8 |
| *IRF7* | Interferon regulatory factor 7 |
| *JUN* | Jun proto-oncogene, AP-1 transcription factor subunit |
| *KCNJ16* | Potassium two pore domain channel subfamily K member 7 |
| *KCNK7* | Potassium two pore domain channel subfamily K member 7 |
| *KCNQ1* | Potassium voltage-gated channel subfamily Q member 1 |
| *KEGG* | 1.1.2 Kyoto encyclopedia of genes and genomes |
| *LAT1* | Large neutral amino acid transporter 1 |
| LDA | Linear Discriminant Analysis |
| LEfSE | Linear Discriminant Analysis Effect Size |
| *NaPi-IIb* | Sodium-dependent phosphate transport protein 2b |
| NCBI | National Center for Biotechnology Information |
| PDR | Protein deposition ratio |
| *PEPT1* | Polypeptide transporter 1 |
| *PEPT2* | Polypeptide transporter 2 |
| PER | Protein efficiency ratio |
| qPCR | Real-time quantitative polymerase chain reaction |
| RPS18 | Ribosomal protein S18 |
| SAR | Sequence read archive |

| | |
|---|---|
| SGR | Specific growth rate |
| *SLC30A1* | Solute carrier family 30 member 1 |
| *SLC41A3* | Solute carrier family 41 member 3 |
| *SLC52A3* | Solute carrier family 52 member 3 |
| SR | Survival rate |
| *STAT1* | Signal transducer and activator of transcription 1 |
| *TBX21* | T-box transcription factor 21 |
| *TGF-β2* | Transforming growth factor beta 2 |
| *TMEM37* | Transmembrane protein 37 |
| *TNF-α* | Tumor necrosis factor alpha |
| *TRPV6* | Transient receptor potential cation channel subfamily V member 6 |
| VSI | Viscerosomatic index |
| WGR | Weight gain rate |
| $Y_2O_3$ | Yttrium trioxide |

## 2. Supplementary Figures

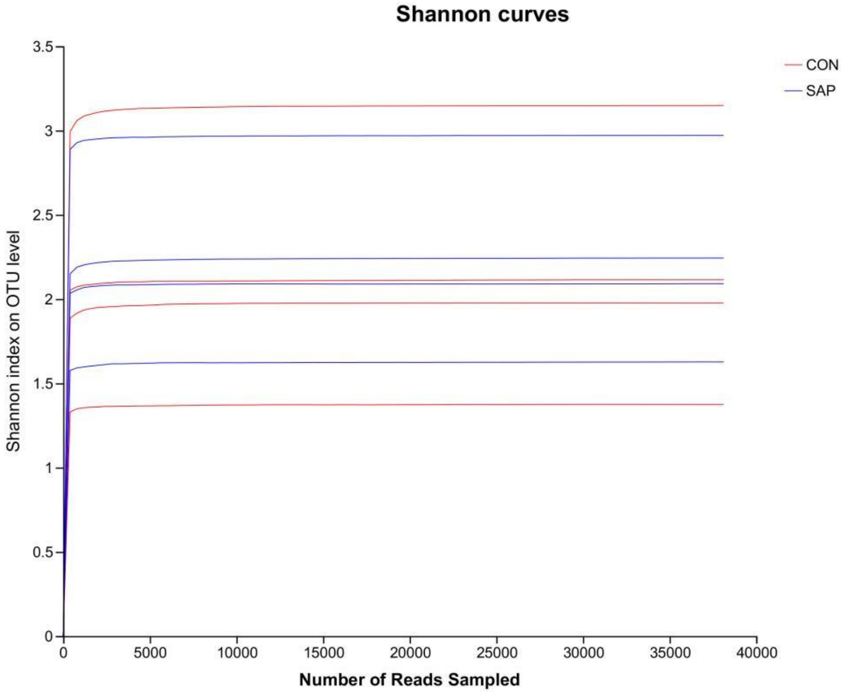


**Supplementary Figure S1. Rarefaction curves of intestinal microbiota（n=4）**

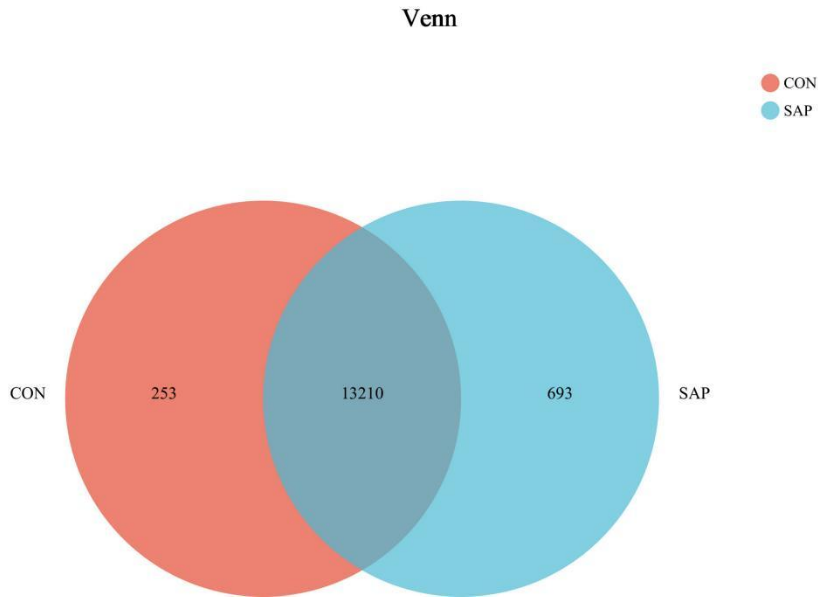


**Supplementary Figure S2. Venn diagram of transcriptome expressed genes (n=3)**

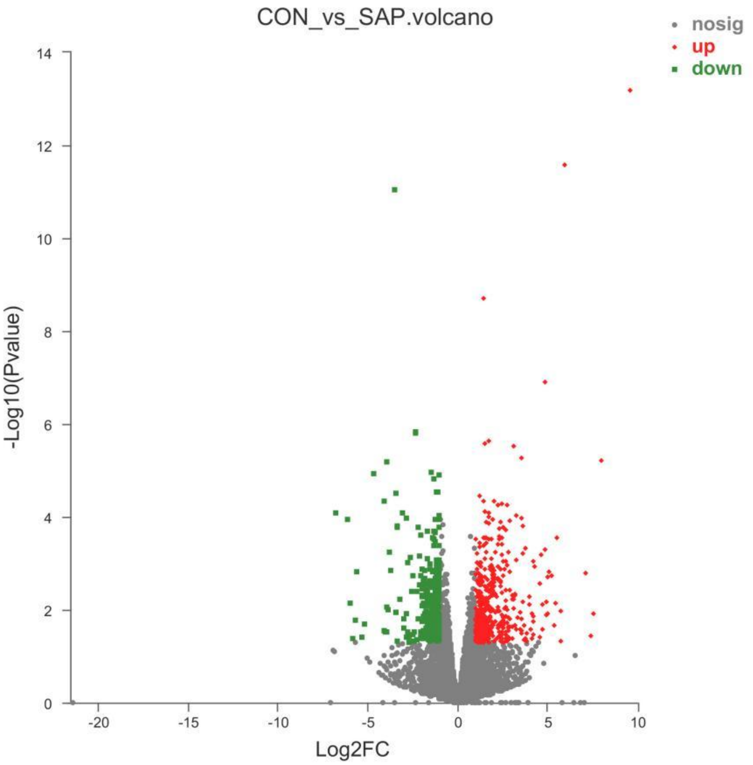


**Supplementary Figure S3. Volcano Plot of DEGs (n=3)**
